# Discovery of CRBN-recruiting PROTAC degraders of the METTL3-METTL14 complex

**DOI:** 10.1007/s00044-025-03464-8

**Published:** 2025-09-05

**Authors:** Alexis R. Smith, Rukiye Nar, Yafang Li, Abhishek Gour, Abhisheak Sharma, Zhijian Qian, Guangrong Zheng, Zhixing Wu

**Affiliations:** 1 University of Florida, College of Pharmacy, Department of Medicinal Chemistry, Gainesville, FL USA; 2 University of Florida, College of Medicine, Division of Hematology & Oncology, Gainesville, FL USA; 3 University of Florida, College of Pharmacy, Department of Pharmaceutics, Gainesville, FL USA

**Keywords:** Targeted protein degradation, METTL3-METTL14 degrader, Anticancer, AML

## Abstract

METTL3 and METTL14, key components of the m^6^A writer complex, are frequently overexpressed in various malignancies, including acute myeloid leukemia (AML), where aberrant methylation has been linked to the upregulation of oncogenic transcription. Therefore, targeting the METTL3/METTL14 complex represents a potential therapeutic approach for AML. Although several METTL3 inhibitors have been discovered, their SAM-competitive mode of action often results in reduced cellular potency, prompting interest in alternative strategies such as targeted protein degradation. In this article, we expand upon reported METTL3/METTL14 complex degraders through exploration of CRBN-recruiting proteolysis-targeting chimeras (PROTACs) from multiple exit vectors of UZH2, a reported METTL3 inhibitor. The most potent PROTAC, **4j**, demonstrated sub-micromolar degradation potency in MV4.11 cells with DC_50_ values of 0.44 µM for METTL3 and 0.13 µM for METTL14. Notably, **4j** showed enhanced cytotoxicity in MV4.11 cells compared to well-validated METTL3 inhibitors, underscoring the therapeutic potential of targeted degradation of the METTL3/METTL14 complex in AML.

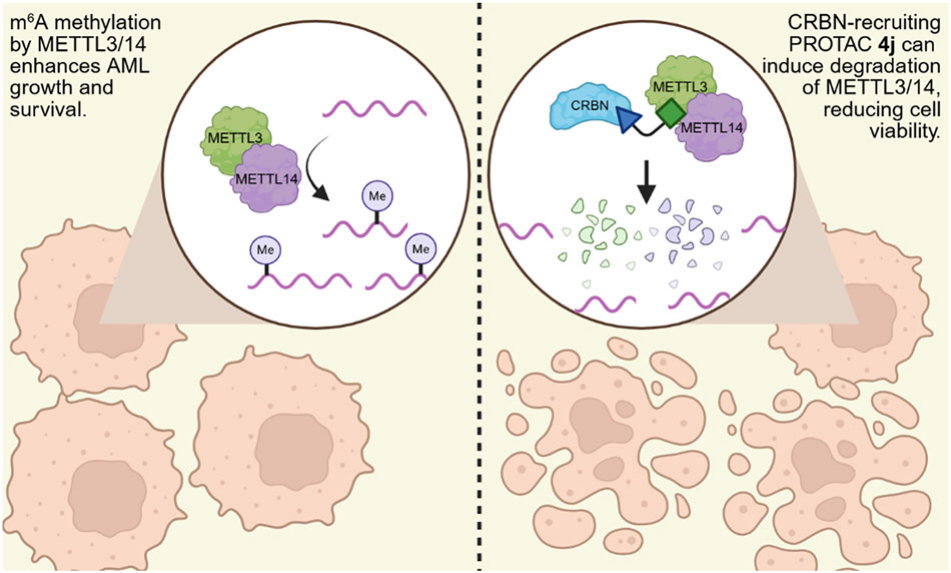

## Introduction

With the discovery of reversible modifications on RNA and the responsible proteins in recent years, a new field of study has emerged, attempting to harness the epitranscriptomic control of gene expression in the cell. Enzymatic post-transcriptional modification of RNA is an internal mechanism for tight control of gene expression, affecting the stability, translocation, and translation of mRNA [1]. In a normal cell, the balance between the modified and unmodified RNAs is maintained to promote typical cellular activities; however, disruption of this homeostasis leads to irregular modifications that result in diseases, including the development and progression of certain cancers [2]. The most characterized and prominent modification of mRNA is *N*^6^-methyladenosine (m^6^A), which plays key roles in RNA metabolism and maintaining proper RNA functions, including nuclear export, processing, splicing, and translation [3]. Therefore, it is not surprising that many cancers, such as acute myeloid leukemia (AML), lymphoma, lung cancer, and glioblastoma, have been found to have abnormal levels of m^6^A [4, 5].

The m^6^A modification often occurs in substrate RNA with a GGACU sequence, commonly near the 3′ and 5′ untranslated regions of mRNAs and stop codons [6, 7]. Many proteins are associated with this modification as it is reversible, allowing for precise control of altered RNA and the cascading effects thereof [3]. The involved proteins can be split into three classes based on their effects: the ‘writer’ proteins, which install the modification, ‘reader’ proteins, which can recognize the methylation and recruit other proteins for more specific effects, and ‘eraser’ proteins, which remove the modification and return the RNA to its unaltered state [8]. Methyltransferase-like protein 3 (METTL3), in complex with METTL14 (methyltransferase-like protein 14), acts as the most prevalent and well-studied catalytic ‘writer’ unit of the m^6^A methyltransferase complex and is responsible for almost all m^6^A modifications on mRNA [9]. The METTL3-METTL14 heterodimer acts as part of a larger methyltransferase-associated complex (MACOM) to catalyze the methylation of mRNA using *S*-adenosylmethionine (SAM) as the methyl donor molecule, with METTL3 functioning as the catalytic unit and METTL14 required for RNA binding leading to m^6^A deposition [3]. While METTL3 appears to act as a tumor suppressor in a few cancers [10–12], many reports suggest that METTL3 functions as an oncogene in numerous cancer types [2, 4, 13–17]. In addition, research on human cancer cell lines and mouse models has established METTL3 as a potential novel anticancer target [18].

**Fig. 1:**
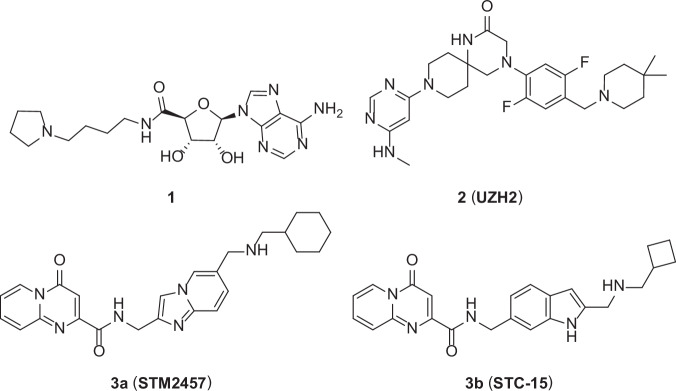
Chemical structures of selected SAM-competitive METTL3 inhibitors

To date, three types of SAM-competitive small molecule inhibitors of METTL3 have been disclosed (Fig. 1) [18]. In 2020, Caflisch’s group discovered compound **1**, an adenine derivative, which inhibited METTL3 activity with an IC_50_ of 8.7 µM in the reader-based HTRF assay [19]. However, adenosine analogs frequently face issues as potential therapeutic agents because of their inadequate cellular permeability and the tendency to bind promiscuously to other SAM-utilizing enzymes. The follow-up medicinal chemistry efforts focused on the non-adenosine chemotypes from the same group led to the discovery of UZH2 (**2**), which displayed potent METTL3 inhibition with an IC_50_ of 5 nM in enzyme-based assays, excellent cell permeability, and moderate metabolic stability [20]. In 2021, Storm Therapeutics reported another non-adenosine METTL3 inhibitor STM2457 (**3a**), which showed potent METTL3/14 catalytic activity inhibition with an IC_50_ of 16.9 nM and a high METTL3/14 binding affinity (*K*_d_) of 1.4 nM [21]. STM2457 demonstrated an anti-leukemic effect in human AML patient-derived xenografts (PDX) models and a primary mouse MLL-AF9/Flt3^Itd/+^ model. Despite their potent enzymatic inhibition against METTL3, much higher concentrations of STM2457 and UZH2 (IC_50_s at 1.52 µM (HeLa cells) and 0.85 µM (MOLM-13 cells), respectively) were required to achieve anti-proliferative effects in human AML cell lines and reduction of m^6^A/A in polyadenylated RNA in MOLM-13 cells. This reduced cellular effect may be attributed to the high intracellular concentration of SAM and *S*-adenosyl homocysteine (SAH) [20, 21]. Therefore, discovering new METTL3 modulators is highly desirable. While small molecule inhibitors of METTL3 continue to be developed, and a more recent compound from Storm Therapeutics (STC-15, **3b**) is currently in clinical trials, alternative strategies such as proteolysis-targeting chimeras (PROTACs) offer a promising approach to modulate the METTL3-METTL14 complex.

PROTACs are molecules that incorporate a warhead moiety that binds the protein of interest (POI) and an E3 ligase ligand, connected through an appropriate linker. These compounds facilitate the polyubiquitination of the POI by bringing it to the vicinity of E3 ligases, ultimately leading to the degradation of POI by the ubiquitin-proteasome system (UPS) [22]. Some of the innate advantages of PROTACs are evident when considering their application to METTL3 degradation. First, PROTACs catalytically induce protein degradation in a sub-stoichiometric manner, so a much lower concentration is needed compared to METTL3 inhibitors that rely on equilibrium occupancy. The event-driven pharmacology of PROTACs is also particularly suited to overcoming competition from the endogenous ligands SAM and SAH. Reduced concentration is important to provide a clinically meaningful therapeutic effect, and can also significantly reduce off-target, dose-limiting toxicities. In addition, PROTACs can deplete both the catalytic and non-catalytic functions of the target proteins. METTL3 is catalytically a m^6^A methyltransferase, but it is also pivotal in promoting the translation of a subset of mRNAs through interaction with eukaryotic initiation factor 3 subunit h (eIF3h), many of which are oncogenic [23].

The development of PROTACs for METTL3/14 has just begun to emerge, with several publications in 2024 [24–27]. These publications, including our efforts, have reported PROTACs using either a von Hippel-Lindau (VHL) E3 ligase ligand or thalidomide as a cereblon (CRBN) ligand, connected to the pyrimidine end of the reported METTL3 inhibitor UZH2 with alkane-chain linkers or PEG-chain linkers. Herein, we expand upon reported structures in the literature thus far with novel CRBN-based PROTAC degraders targeting the METTL3-METTL14 complex, varying the CRBN ligand used and exploring alternate linkage strategies. The optimal compound **4j** (**ZW30441**) showed potent degradation activity against METTL3 and METTL14 with DC_50_ values of 0.44 µM and 0.13 µM, respectively, in the human AML MV4-11 cell line.

## Results and discussion

### Design of METTL3 PROTAC degraders

**Fig. 2:**
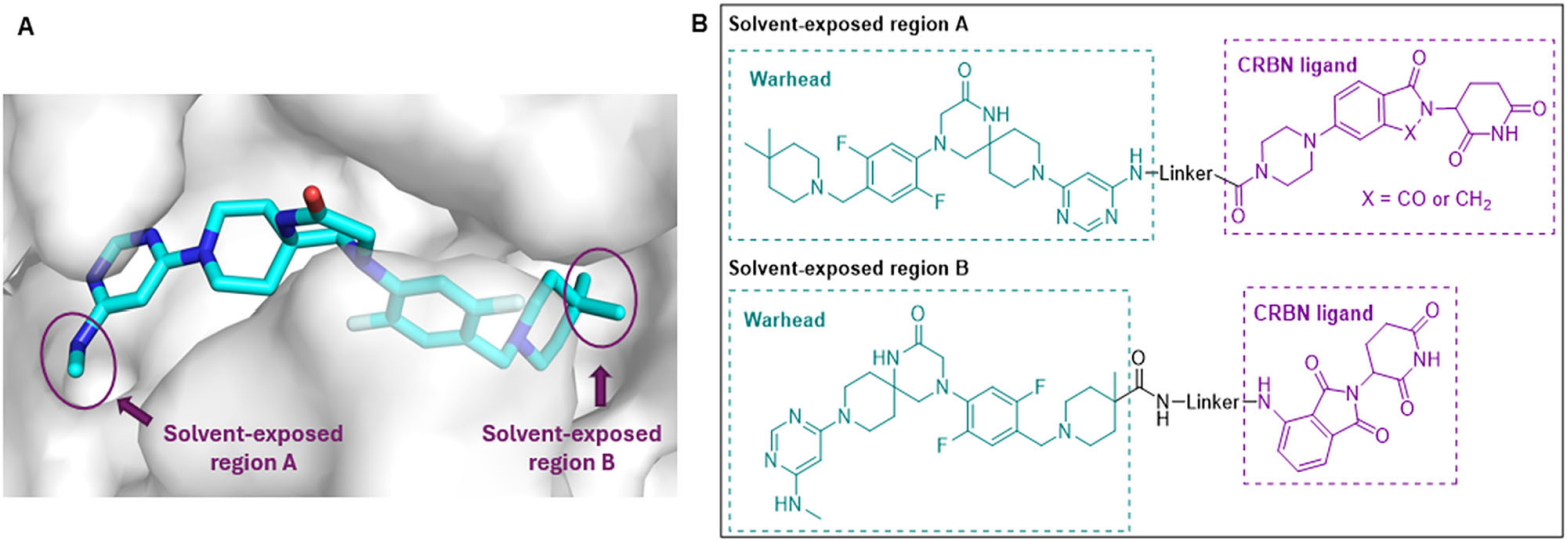
Design of METTL3 PROTAC degraders. **A** Structural analysis of UZH2 in METTL3 binding site (PDB: 7O2F). The arrows indicate the linker vectors for the PROTAC design. **B** Chemical structures of designed METTL3 PROTACs targeting solvent exposure regions A and B

We chose to utilize UZH2 as the warhead in our development of METTL3 PROTAC degraders, since UZH2 is a selective and potent METTL3 inhibitor with moderate metabolic stability, and the structure-activity relationship (SAR) studies of UZH2 have been systematically discussed [20]. The analysis of the crystal structure of UZH2 in the METTL3 binding site revealed that the methylamine on the pyrimidine extended to the solvent-exposed region A (Fig. 2A). The reported SAR studies on UZH2 showed that methylamine can be replaced by a benzylamine moiety [20], indicating the methyl group is a suitable position for linker attachment. Furthermore, the 4,4-dimethylpiperidine moiety of UZH2 is relatively deep in the binding pocket, but one of the methyl groups projects into the solvent-exposed region B (Fig. 2A), thus presenting another vector for linker conjugation. Based on these observations, we designed two series of METTL3 degraders by tethering UZH2 to thalidomide or lenalidomide for CRBN E3 ligase recruiting through a set of alkane-chain linkers (Fig. 2B).

### Structure-degradation relationship study of METTL3 degraders

Based on the design strategy discussed above, we first obtained a set of METTL3 degraders (**4a**-**4f**) by modifying the methylamine of UZH2 to connect to the thalidomide ligand via alkane-chain linkers of different lengths (Table 1). The degradation potency was assessed by immunoblotting assays in MV4-11 cells after treatment with compounds for 24 h. The results showed that compound **4d**, containing a seven-carbon linker, induced METTL3 degradation by 68 and 52% after the treatment at 1 µM and 0.3 µM, respectively (Table 1). Interestingly, **4d** was also able to reduce METTL14 levels by 67 and 51% at 1 µM and 0.3 µM, respectively. Furthermore, either shortening (**4a-4c**) or extending (**4e-4f**) the linker length by 1-3 carbon(s) led to reduced degradation activities toward METT3 and METT14 (Table 1).

**Table 1:** Degradation Potency of Compounds **4a**-**4k**^a^


Compound	n	X	R	% reduction in MV4-11 cells			
				1.0 µM		0.3 µM	
				METTL3	METTL14	METTL3	METTL14
**4a**	1	CO	H	47	27	14	6
**4b**	2	CO	H	56	58	45	36
**4c**	3	CO	H	62	61	43	46
**4d**	4	CO	H	68	67	52	51
**4e**	5	CO	H	52	53	38	39
**4f**	6	CO	H	nd^*b*^	nd	nd	nd
**4g**	3	CH_2_	H	25	34	9	0
**4h**	4	CH_2_	H	69	64	46	40
**4i**	5	CH_2_	H	73	72	52	54
**4j** (**ZW30441**)	6	CH_2_	H	81	77	58	56
**4k** (**ZW30441NC**)	6	CH_2_	Me	nd	nd	nd	nd

^*a*^Degradation potency was determined by immunoblotting after treatment with compounds in MV4-11 cells for 24 h

^*b*^No degradation (nd)

Next, we synthesized a second series of degraders **4g-4j** by connecting the methylamine moiety of UZH2 to lenalidomide (Table 1), a thalidomide derivative known to confer distinct neosubstrate selectivity through CRBN recruiting [28]. Within this subset, degradation potency against METTL3 and METTL14 varied with linker length. Among them, **4j** (ZW30441), carrying a nine-carbon linker, exhibited potent METTL3 degradation with 81 and 58% reduction at 1 µM and 0.3 µM, respectively, and showed comparable degradation activity against METTL14. We also synthesized compound **4k** (ZW30441NC) as a negative control, in which the glutarimide moiety of lenalidomide was methylated to inactivate CRBN binding. As expected, ZW30441NC did not induce degradation of either METTL3 or METTL14.

In addition, we synthesized another set of METTL3 degraders (**5a-5d**) by conjugating one of the methyl groups from the 4,4-diemthylpieridine moiety of UZH2 to the thalidomide ligand via alkane-chain linkers of varying lengths (Table 2). Unfortunately, while there was a noticeable degradation observed, none of these compounds induced above 60% degradation against METTL3 or METTL14. We thus continued to characterize our lead compound from the previous series.

**Table 2:** Degradation Potency of Compounds **5a**-**5d**^a^


Compound	n	% degradation in MV4-11 cells			
		1.0 µM		0.3 µM	
		METTL3	METTL14	METTL3	METTL14
**5a**	1	52	21	33	8
**5b**	3	56	21	55	20
**5c**	5	54	24	50	20
**5d**	7	3	21	26	34

^*a*^Degradation potency was determined by immunoblotting after treatment with compounds in MV4-11 cells for 24 h

### Biological characterization of ZW30441

**Fig. 3:**
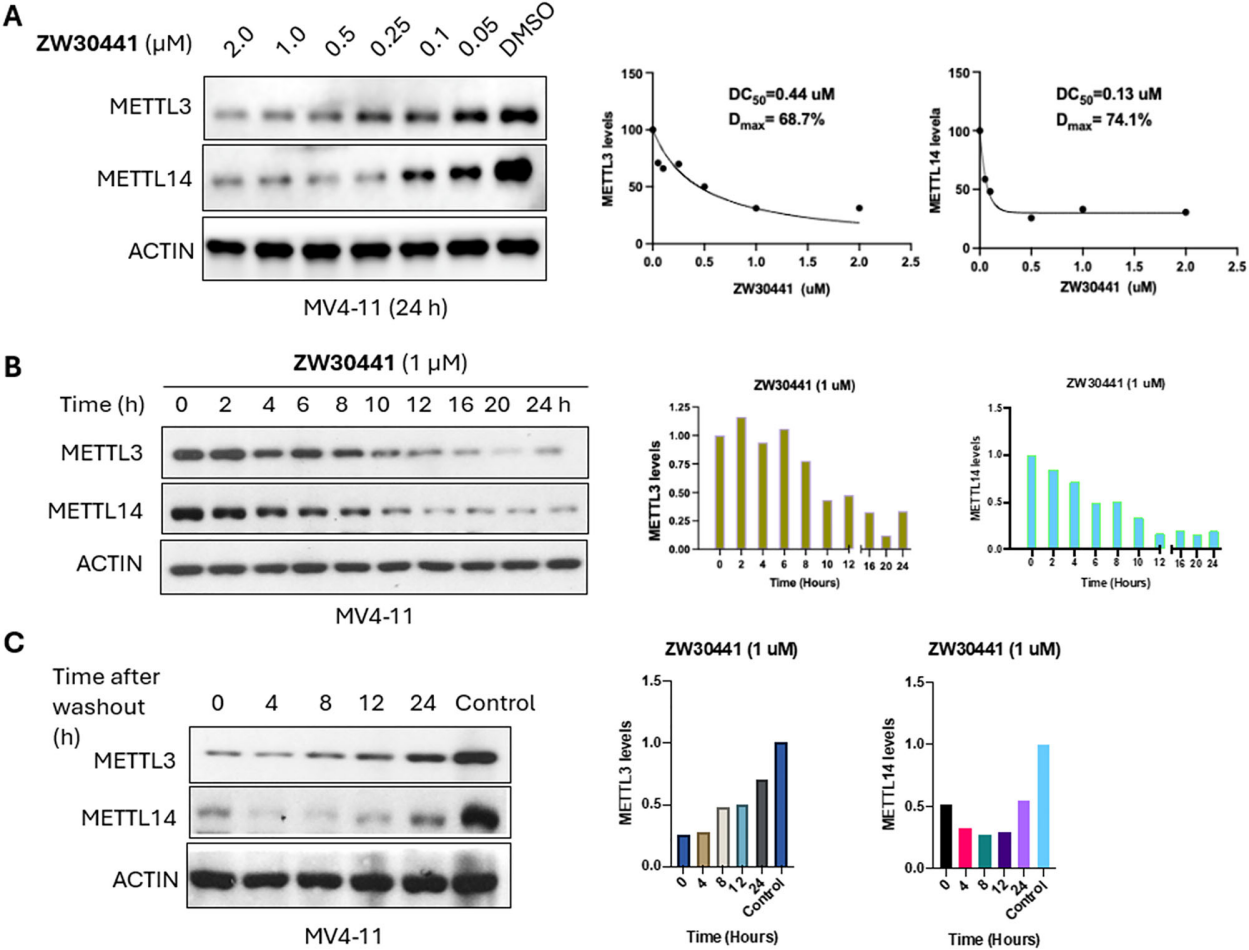
ZW30441 induced degradation of METTL3 and METTL14 in a dose- and time-dependent manner. **A** Western blots showed the levels of the METTL3 and METTL14 protein in MV4-11 cells after the treatment of ZW30441 at the indicated concentrations for 24 h (left). METTL3 and METTL14 protein levels were normalized against ACTIN and plotted for DC_50_ and D_max_ determination (right). **B** The levels of METTL3 and METTL14 protein in MV4-11 cells were analyzed by Western blots after the treatment of 1 µM of ZW30441 for various durations. **C** METTL3 and METTL14 protein levels were analyzed by Western blots in MV4-11 cells after treatment with 1 µM of ZW30441 for 20 h followed by drug withdrawal and cultured without ZW30441 for 0 to 24 h, as indicated

ZW30441 emerged as the most potent degrader of METTL3 and METTL14 in the initial two-concentration screening. To further characterize this compound, MV4-11 AML cells were treated with ZW30441 across a range of concentrations for 24 h. The results showed that ZW30441 dose-dependently induced METTL3 and METTL14 degradation, with DC_50_ values of 0.44 µM and 0.13 µM, and D_max_ values of 80 and 65%, respectively (Fig. 3A). Time-course experiments at 1 µM revealed that ZW30441 induced gradual degradation of METTL3 and METTL14, with half-lives (t_1/2_) of 9.0 and 8.0 h, respectively, and maximum degradation observed at 20 h (Fig. 3B). We next performed a washout experiment following 20 h of treatment of MV4-11 cells with 1 µM of ZW30441, which showed that ZW30441-induced degradation of the METTL3-METTL14 complex lasted for around 24 h after the removal of ZW30441 from the culture media (Fig. 3C). Collectively, these results indicate that ZW30441 effectively and durably induces degradation of the METTL3-METTL14 complex in a dose- and time-dependent fashion.

**Fig. 4:**
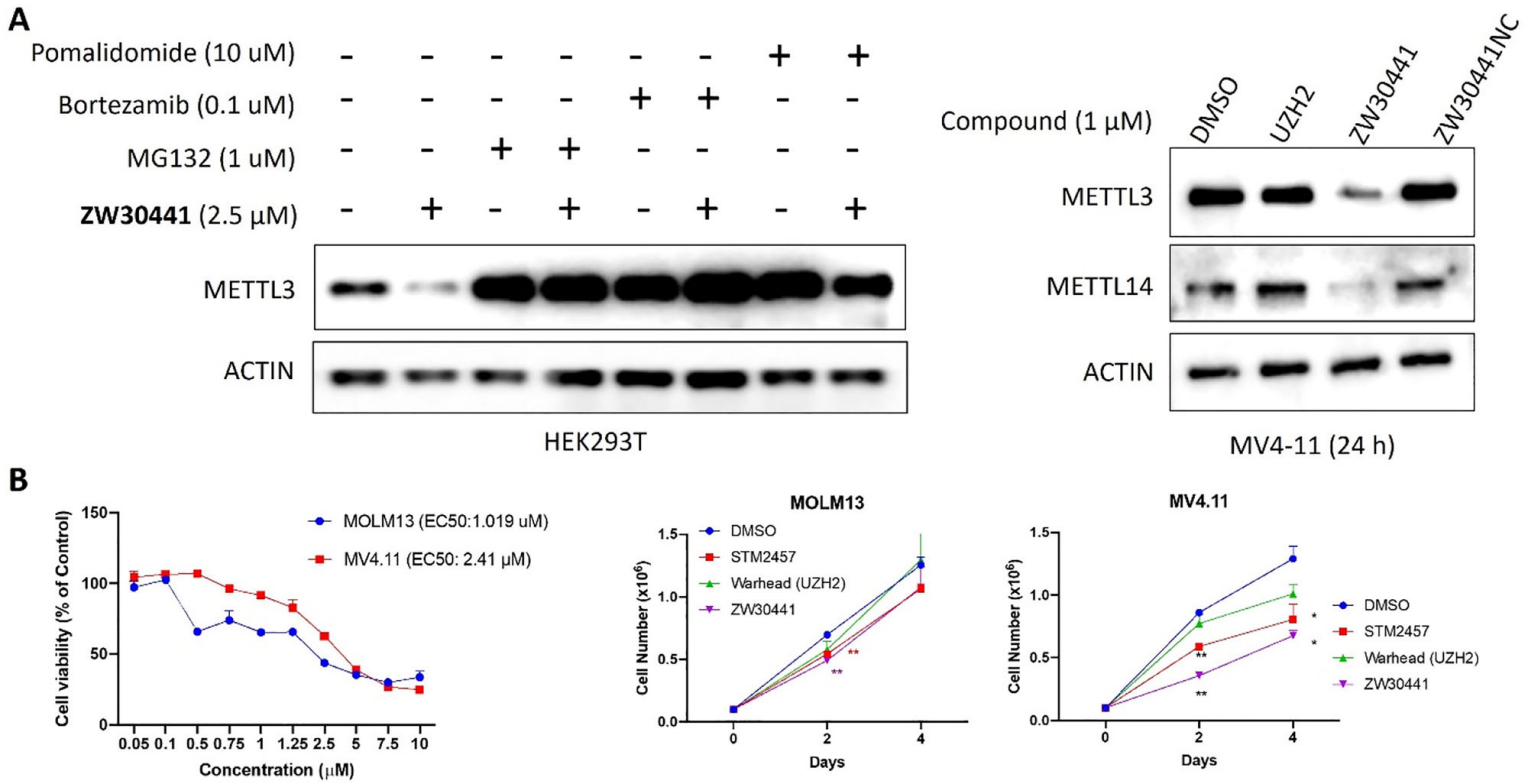
ZW30441 induced METTL3-METTL14 complex degradation through the ubiquitin-proteasome system and affected the survival of human AML cell lines. **A** Western blot analysis of METTL3 expression in HEK293T cells after pretreatment with the CRBN ligand pomalidomide (10 µM) or the proteasome inhibitors bortezamib (0.1 µM) or MG132 (1 µM) for 2 h, followed by treatment with ZW30441 (2.5 µM) for 24 h (left). Western blot analysis of METTL3 and METTL14 levels in MV4-11 cells after treatment with UHZ2, ZW30441, and the negative control ZW30441NC at the indicated concentration for 24 h (right). **B** Cell viability study of ZW30441 in MOLM13 and MV4-11 cell lines (left). Cell proliferation study of ZW30441 (5 µM) in MOLM13 and MV4-11 cells with comparison to STM2457 and UZH2 (right). EC₅₀ values were calculated by normalizing viability data to vehicle-treated controls

We next explored the mechanisms of ZW30441-induced METTL3-METTL14 degradation. HEK293T cells were pretreated with the proteasome inhibitors bortezomib (0.1 µM) or MG132 (1 µM) for 2 h before ZW30441 (2.5 µM) administration (Fig. 4A). The results showed that the proteasome inhibitors effectively blocked the METTL3 degradation induced by ZW30441. In addition, we observed that the degradation activity of ZW30441 on METTL3 was significantly reduced when HEK293T cells were pretreated with a CRBN ligand (pomalidomide, 10 µM). It was also demonstrated that MV4-11 cells were not degraded upon treatment with UZH2 (1 µM) or the negative control ZW30441NC (1 µM), indicating that degradation required the binding of ZW30441 to both METTL3 and CRBN (Fig. 4A). Taken together, these observations confirmed that ZW30441 degrades METTL3 via the proteasome only when both the warhead and CRBN ligand are actively involved, demonstrating the necessity of the ternary complex formation for activity.

METTL3 inhibitors STM2457 and UHZ2 have displayed potent METTL3/14 catalytic activity inhibition and exerted anti-proliferation effects against AML cells. We proceeded to evaluate the cytotoxic activities of ZW30441 in AML cell lines by treating MOLM13 and MV4-11 cells with ZW30441 in increasing doses for 72 h. The results showed that ZW30441 was able to suppress the viability of MOLM13 and MV4-11 cells in a dose-dependent manner with EC_50_ values of 1.02 µM and 2.14 µM, respectively (Fig. 4B). Compared to METTL3 inhibitors STM2457 and UZH2, which also require micromolar concentrations for cellular activity, ZW30441 demonstrated comparable cell growth inhibition in the MOLM-13 cell line and more potent anti-proliferation activity in the MV4-11 cells (Fig. 4B).

To evaluate the potential for advancing this compound to in vivo studies, the in vitro metabolic stability of this compound was determined in human liver microsomes. ZW30441 exhibited a half-life (T_1/2_) of 4.6 ± 0.7 min and an intrinsic clearance (Cl_int_) of 152.1 ± 26.1 uL/min/mg of protein, demonstrating relatively poor metabolic stability. This is not unanticipated for a PROTAC containing a flexible linker, as it has been shown that many rotatable bonds negatively impact the physicochemical properties of a compound, with a suggested guideline of fewer than 14 rotatable bonds proposed to be optimal in the design of a potentially orally bioavailable PROTAC [29, 30]. The flexible linker could then be one potential reason for overall metabolic instability. With this knowledge in hand, further optimization of the lead compound would be necessary before beginning in vivo studies. One potential approach is to introduce more rigid linker systems, thereby reducing molecular flexibility to enhance the metabolic stability while continuing to improve potency.

## Chemistry

**Scheme 1:**
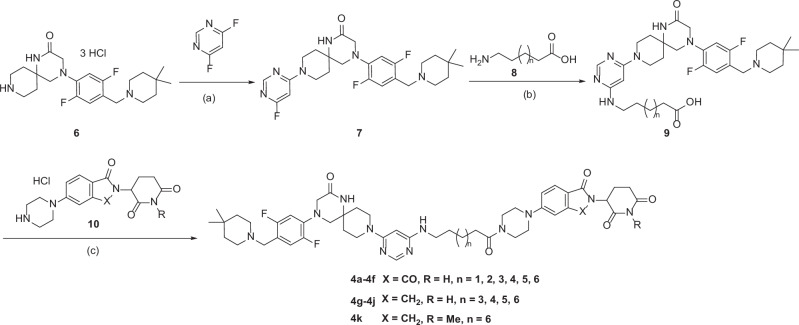
Synthesis of Compounds **4a-4k**^*a*^. ^*a*^Reagents and conditions: (a) 4,6-difluoropyrimidine, DIPEA, isopropanol, 80 °C, microwave irradiation, 3 h; (b) **8**, DIPEA, isopropanol, H_2_O, 130 °C, microwave irradiation, 5 h; (c) **10**, HATU, DMF, DIPEA, 25 °C, 2 h

The synthesis of compounds **4a-4k** is depicted in Scheme 1. The starting material **6** was synthesized according to the method reported by the Caflisch’s group [20]. A nucleophilic aromatic substitution (SnAr) reaction between intermediate **6** and 4,6-difluoropyrimidine afforded the fluoride intermediate **7** in quantitative yield. Then, **7** underwent another SnAr reaction with ω-aminoalkylcarboxylic acid **8** in an isopropanol/H_2_O (1:1) solution, giving the acid intermediate **9** after trituration with MeOH and DCM. Finally, a HATU-mediated amide coupling of the CRBN ligand **10** with **9** in DMF gave the desired compounds **4a-4k**.

**Scheme 2:**
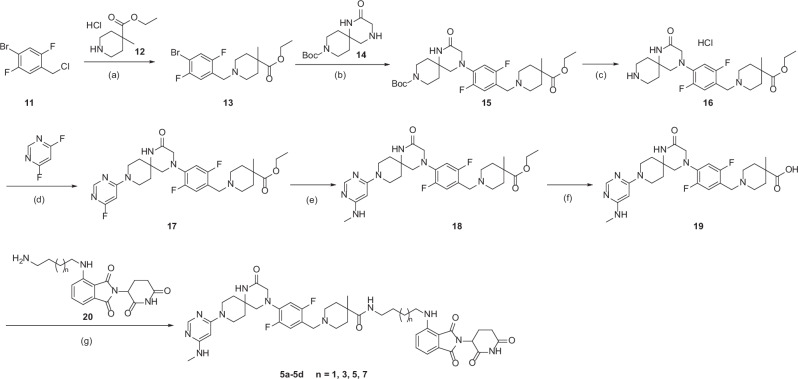
Synthesis of Compounds **5a-5d**^*a*^. ^*a*^Reagents and conditions: (a) **12**, K_2_CO_3_, DMF, 50 °C, 12 h; (b) **14**, RuPhos-Pd-G4, RuPhos, Cs_2_CO_3_, 1,4-dioxane, 110 °C, 48 h; (c) conc. HCl, MeOH, rt, 18 h; (d) 4,6-difluoropyrimidine, DIPEA, isopropanol, 80 °C, microwave irradiation, 3 h; (e) MeNH_2_, 130 °C, microwave irradiation, 1 h; (f) 2 N NaOH, THF, MeOH, 60 °C, 18 h; (g) **20**, HATU, DIPEA, DMF, rt, 30 min

The synthetic strategy for compounds **5a-5d** is depicted in Scheme 2. Alkylation of ethyl 4-methylpiperidine-4-carboxylate hydrochloride (**12**) with 4-bromo-2,5-difluorobenzyl chloride (**11**) gave bromide **13**. Buchwald-Hartwig coupling of **13** with spiroamine **14**, followed by Boc deprotection with HCl in MeOH, produced intermediate **16**. Consecutive S_N_Ar reactions with 4,6-difluoropyrimidine and MeNH_2_ under microwave irradiation afforded ester intermediate **18**, which was hydrolyzed with 2 *M* NaOH in THF and MeOH to give the acid intermediate **19**. Subsequent HATU-mediated amide coupling between **19** and the CRBN ligand **20** successfully delivered compounds **5a-5d**.

## Conclusion

In this study, we report the development of CRBN-recruiting PROTACs targeting the METTL3-METTL14 complex. We evaluated the influence of E3 ligase ligands by comparing two commonly used CRBN ligands. The lenalidomide-based PROTAC **4j** (ZW30441) exhibited the most potent activity, achieving degradation of METTL3 with a D_max_ of 80% and a DC_50_ of 0.44 µM in MV4-11 cells. ZW30441 also effectively degraded METTL14. Its activity was abrogated by proteasome inhibitors and by co-treatment with pomalidomide, verifying the PROTAC mechanism of action. A novel linkage site was also explored with compounds **5a-5d**; however, these analogs showed lower activity, indicating that this alternative conjugation site may be less favorable, despite the potential for further optimization. Due to the compound’s quick metabolism and fast clearance, a second-generation of degraders with more rigid linkers will need to be evaluated to improve compound stability before in vivo investigation can be done. Overall, the lead compound ZW30441 demonstrated robust, CRBN and proteasome-dependent degradation and superior cellular activity compared to the known METTL3 inhibitors UZH2 and STM2547 in MV4-11 cells, and this compound serves as a potent lead compound for further development to increase activity for in vivo study.

## Supplementary information

Supplemental Information

## Data Availability

No datasets were generated or analysed during the current study.
