# Supplementary material for: Discovery of CRBN-recruiting PROTAC degraders of the METTL3-METTL14 complex
Source: Med Chem Res. 2025 Sep 5;34(11):2299–308. doi: 10.1007/s00044-025-03464-8 (PMC12615518; doi:10.1007/s00044-025-03464-8)
Supplement: Supplementary file 1 — Supplemental Information [file 44_2025_3464_MOESM1_ESM.docx]

Supplemental Information – Table of Contents

**1. Experimental methods**

1.1 General Chemistry Methods ………………………………………………………….1

1.2 Scheme for Synthesis of Compounds 4a-4k…………………………………………. 2

1.3 Synthesis of Compounds 4a-4k……………………………………………………….2

1.4 Scheme for Synthesis of Compounds 5a-5d…………………………………………16

1.5 Synthesis of Compounds 5a-5d……………………………………………………...17

1.6 Biological Methods.………………………………………………………………….25

**2. Compound spectra**

2.1 NMR spectra for reported compounds……………………………………………….27

2.2 HRMS data for final compounds…………………………………………………….48

**1. Experimental methods**

**1.1 General Chemistry Methods**.

All solvents and reagents were used as purchased from commercial suppliers unless otherwise stated. Anhydrous solvents were obtained commercially or dried using a solvent purification system, filtering through two columns of activated alumina and 4 Å molecular sieves, respectively. Reactions needing anhydrous and inert conditions were performed under an atmosphere of Nitrogen. Thin-layer chromatography was performed on silica-coated glass plates, with visualization of compounds under UV light at 254 nm or with iodine vapor staining. Products were purified by flash column chromatography on silica gel (230-400 mesh) using a Biotage Selekt system. ^1^H and ^13^C spectra were taken on a Brüker 600 instrument using deuterated solvents with an internal TMS standard. Chemical shifts are reported in parts per million (ppm) and calibrated using residual solvent signal (Chloroform-*d* = CDCl_3_, 7.26 ppm for ^1^H NMR and 77.0 ppm for ^13^C NMR; Methanol-*d4* = CD_3_OD, 3.31 ppm for ^1^H NMR and 49.0 for ^13^C NMR). Reported multiplicities include: singlet (s); broad singlet (br s); doublet (d); triplet (t); quartet (q); pentet (p); multiplet (m). Coupling constants (*J*) are reported in Hertz (Hz). ESI mass spectra were recorded on an Advion AVANT LC system, and mass-to-charge ratios (*m/z*) are reported.

**1.2 Scheme for Synthesis of Compounds 4a-4k**

**1.3 Synthesis of Compounds 4a-4k**

To a solution of 4-(4-((4,4-dimethylpiperidin-1-yl)methyl)-2,5-difluorophenyl)-1,4,9-triazaspiro[5.5]undecan-2-one hydrochloride **6** (100 mg, 0.2 mmol) in isopropanol (2.0 mL) in a microwave vial was added 4,6-difluoropyrimidine (46 mg, 0.4 mmol) and DIPEA (129 mg, 1.0 mmol). The microwave vial was capped, and the reaction mixture was heated to 80 °C, stirring for 3 hours. After cooling to room temperature, the reaction mixture was concentrated, and ethyl acetate (20 mL) and aqueous ammonium chloride solution (20 mL) were added. The aqueous phase was extracted with ethyl acetate (3 x 10 mL), and the combined organic layers were washed with brine (30 mL), dried over anhydrous Na_2_SO_4_, filtered, and concentrated under reduced pressure. The resulting residue was purified by silica gel flash column chromatography (100:0 to 12:1 dichlormethane/methanol) to give the desired product **7** (100 mg, 99 %) as an off-white solid.

**^1^H NMR** (600 MHz, CDCl_3_) δ 8.37 (d, *J* = 2.4 Hz, 1H), 7.18 (br s, 1H), 6.64 – 6.58 (m, 2H), 6.07 (s, 1H), 3.88 – 3.81 (m, 2H), 3.76 (s, 2H), 3.75 – 3.70 (m, 2H), 3.54 (s, 2H), 3.33 (s, 2H), 2.46 (s, 4H), 2.04 (ddd, *J* = 11.6, 7.3, 3.8 Hz, 2H), 1.88 (ddd, *J* = 13.0, 7.9, 4.0 Hz, 2H), 1.44 (s, 4H), 0.94 (s, 6H) ppm.

**^13^C NMR** (150 MHz, CDCl_3_) δ 172.15, 170.53, 167.72, 164.69, 164.62, 158.87 (d, *J* = 235.9 Hz), 158.45, 158.33, 150.98 (dd, *J* = 240.4, 3.0 Hz), 118.32 (br s), 105.84 – 105.49 (m), 85.50, 85.26, 56.45 (d, *J* = 2.0 Hz), 53.36, 52.97, 49.74, 40.44, 35.31, 29.71, 28.32 ppm.

**ESI-MS** *m/z* calculated for C_26_H_33_F_3_N_6_O [M+H]^+^: 503.3; found 503.2.

**General Procedure**: To a solution of 4-(4-((4,4-dimethylpiperidin-1-yl)methyl)-2,5-difluorophenyl)-9-(6-fluoropyrimidin-4-yl)-1,4,9-triazaspiro[5.5]undecan-2-one **7** (1 eq) in isopropanol (0.2 mL) and water (0.2 mL) in a microwave vial was added DIPEA (10 eq). Amine **8a-f** (10 eq) was added to the reaction mixture, and the microwave vial was capped. The reaction was heated to 120 °C, stirring for 5 hours. After cooling to room temperature, the solvent was removed through concentration under reduced pressure. Methanol (5 mL) was added, and the mixture was sonicated for 30 minutes before the precipitate was removed via filtration and the filtrate was concentrated under reduced pressure. The resulting products **9a-f** were moved on to the next reaction without further purification.

**9a**: 5-((6-(4-(4-((4,4-dimethylpiperidin-1-yl)methyl)-2,5-difluorophenyl)-2-oxo-1,4,9-triazaspiro[5.5]undecan-9-yl)pyrimidin-4-yl)amino)pentanoic acid

Compound **9a** was synthesized according to the general procedure described above using 5-aminopentanoic acid (**8a**) at a 15.0 mg scale, yielding 30.0 mg.

**ESI-MS** *m/z* calculated for C_31_H_43_F_2_N_7_O_3_ [M+H]^+^: 600.3; found 600.2.

**9b**: 6-((6-(4-(4-((4,4-dimethylpiperidin-1-yl)methyl)-2,5-difluorophenyl)-2-oxo-1,4,9-triazaspiro[5.5]undecan-9-yl)pyrimidin-4-yl)amino)hexanoic acid

Compound **9b** was synthesized according to the general procedure described above using 6-aminohexanoic acid (**8b**) at a 15.0 mg scale, yielding 27.0 mg.

**ESI-MS** *m/z* calculated for C_32_H_45_F_2_N_7_O_3_ [M+H]^+^: 614.4; found 614.4.

**9c**: 7-((6-(4-(4-((4,4-dimethylpiperidin-1-yl)methyl)-2,5-difluorophenyl)-2-oxo-1,4,9-triazaspiro[5.5]undecan-9-yl)pyrimidin-4-yl)amino)heptanoic acid

Compound **9c** was synthesized according to the general procedure described above using 7-aminoheptanoic acid (**8c**) at a 15.0 mg scale, yielding 26.0 mg.

**ESI-MS** *m/z* calculated for C_33_H_47_F_2_N_7_O_6_ [M+H]^+^: 628.4; found 628.4.

**9d**: 8-((6-(4-(4-((4,4-dimethylpiperidin-1-yl)methyl)-2,5-difluorophenyl)-2-oxo-1,4,9-triazaspiro[5.5]undecan-9-yl)pyrimidin-4-yl)amino)octanoic acid

Compound **9d** was synthesized according to the general procedure described above using 8-aminooctanoic acid (**8d**) at a 20.0 mg scale, yielding 40.0 mg.

**ESI-MS** *m/z* calculated for C_34_H_49_F_2_N_7_O_3_ [M+H]^+^: 642.4; found 642.4.

**9e**: 9-((6-(4-(4-((4,4-dimethylpiperidin-1-yl)methyl)-2,5-difluorophenyl)-2-oxo-1,4,9-triazaspiro[5.5]undecan-9-yl)pyrimidin-4-yl)amino)nonanoic acid

Compound **9e** was synthesized according to the general procedure described above using 9-aminononanoic acid (**8e**) at a 20.0 mg scale, yielding 40.0 mg.

**ESI-MS** *m/z* calculated for C_35_H_51_F_2_N_7_O_3_ [M+H]^+^: 656.4; found 656.4.

**9f**: 10-((6-(4-(4-((4,4-dimethylpiperidin-1-yl)methyl)-2,5-difluorophenyl)-2-oxo-1,4,9-triazaspiro[5.5]undecan-9-yl)pyrimidin-4-yl)amino)decanoic acid

Compound **9f** was synthesized according to the general procedure described above using 10-aminodecanoic acid (**8f**) at a 20.0 mg scale, yielding 35.0 mg.

**ESI-MS** *m/z* calculated for C_36_H_53_F_2_N_7_O_3_ [M+H]^+^: 670.4; found 670.4.

**General Procedure**: To a solution of carboxylic acid **9a-f** (1 eq) in DMF (0.2 mL) was added HATU (1.2 eq), and the resulting solution was stirred at room temperature for 15 minutes. A solution of hydrochloride salt **10a-c** (1 eq) and DIPEA (10 eq) in DMF (0.3 mL) was added to the solution prepared above, and the resulting mixture was stirred for 1 hour at room temperature. The reaction mixture was partitioned between ethyl acetate (10 mL) and water (10 mL). The organic layer was separated, washed with brine (10 mL), dried over anhydrous Na_2_SO_4_, filtered, and concentrated under reduced pressure. The resulting residue was purified by preparative TLC (8:1 dichloromethane/methanol) to give the desired products **4a-k**.

**4a**: 5-(4-(5-((6-(4-(4-((4,4-dimethylpiperidin-1-yl)methyl)-2,5-difluorophenyl)-2-oxo-1,4,9-triazaspiro[5.5]undecan-9-yl)pyrimidin-4-yl)amino)pentanoyl)piperazin-1-yl)-2-(2,6-dioxopiperidin-3-yl)isoindoline-1,3-dione

Compound **4a** was synthesized according to the general procedure described above using 5-((6-(4-(4-((4,4-dimethylpiperidin-1-yl)methyl)-2,5-difluorophenyl)-2-oxo-1,4,9-triazaspiro[5.5]undecan-9-yl)pyrimidin-4-yl)amino)pentanoic acid **(9a**) and 2-(2,6-dioxopiperidin-3-yl)-5-(piperazin-1-yl)isoindoline-1,3-dione hydrochloride (**10a**) at a 30.0 mg scale, yielding 6.0 mg (13.0% yield).

**^1^H NMR** (600 MHz, CDCl_3_) δ 9.53 (s, 1H), 8.13 (s, 1H), 7.70 (dd, *J* = 8.6, 2.0 Hz, 1H), 7.23 (d, *J* = 2.4 Hz, 1H), 7.10 (dd, *J* = 12.7, 6.4 Hz, 1H), 7.04 (d, *J* = 8.5 Hz, 1H), 6.92 (s, 1H), 6.58 (dd, *J* = 10.9, 7.1 Hz, 1H), 5.43 (s, 1H), 5.20 (s, 1H), 4.98 – 4.93 (m, 1H), 3.84 – 3.75 (m, 1H), 3.74 – 3.66 (m, 3H), 3.65 – 3.56 (m, 4H), 3.48 (s, 2H), 3.44 – 3.34 (m, 4H), 3.31 – 3.22 (m, 4H), 2.91 – 2.69 (m, 3H), 2.41 (s, 6H), 2.16 – 2.11 (m, 1H), 1.98 – 1.87 (m, 5H), 1.86 – 1.73 (m, 3H), 1.73 – 1.66 (m, 2H), 1.40 – 1.37 (m, 4H), 0.90 (s, 6H) ppm.

**^13^C NMR** (150 MHz, CDCl_3_) δ 171.39, 171.13, 168.94, 167.96, 167.78, 167.13, 163.23, 162.30, 157.73, 157.25 (d, *J* = 243.0 Hz), 155.08, 150.98 (d, *J* = 243.3 Hz), 137.36, 134.28, 125.46, 120.48, 119.37 (br s), 118.43, 118.40, 108.77, 105.67 (dd, *J* = 27.7, 3.0 Hz), 80.84, 56.65 (d, *J* = 4.3 Hz), 54.66, 53.47, 52.99, 49.74, 49.30, 47.58, 47.45, 44.80, 40.83, 40.25 (d, *J* = 14.1 Hz), 38.54, 35.24 (d, *J* = 19.1 Hz), 32.62, 31.50, 28.91, 28.35, 22.81, 22.40 ppm.

**HRMS-ESI** *m/z* calculated for C_48_H_59_F_2_N_11_O_6_ [M+H]^+^: 924.4618; found 924.4679.

**4b**: 5-(4-(6-((6-(4-(4-((4,4-dimethylpiperidin-1-yl)methyl)-2,5-difluorophenyl)-2-oxo-1,4,9-triazaspiro[5.5]undecan-9-yl)pyrimidin-4-yl)amino)hexanoyl)piperazin-1-yl)-2-(2,6-dioxopiperidin-3-yl)isoindoline-1,3-dione

Compound **4b** was synthesized according to the general procedure described above using 6-((6-(4-(4-((4,4-dimethylpiperidin-1-yl)methyl)-2,5-difluorophenyl)-2-oxo-1,4,9-triazaspiro[5.5]undecan-9-yl)pyrimidin-4-yl)amino)hexanoic acid (**9b**) and 2-(2,6-dioxopiperidin-3-yl)-5-(piperazin-1-yl)isoindoline-1,3-dione hydrochloride (**10a**) at a 27.0 mg scale, yielding 16.0 mg (42.6% yield).

**^1^H NMR** (600 MHz, CDCl_3_) δ 10.13 (s, 1H), 8.11 (s, 1H), 7.68 (d, *J* = 8.4 Hz, 1H), 7.24 (d, *J* = 2.3 Hz, 1H), 7.18 (s, 1H), 7.09 (dd, *J* = 12.8, 6.5 Hz, 1H), 7.01 (dd, *J* = 8.6, 2.3 Hz, 1H), 6.57 (dd, *J* = 10.8, 7.1 Hz, 1H), 5.38 (s, 1H), 5.31 (s, 1H), 4.94 (dd, *J* = 12.4, 5.4 Hz, 1H), 3.83 – 3.72 (m, 2H), 3.72 – 3.52 (m, 8H), 3.48 (s, 2H), 3.43 – 3.38 (m, 4H), 3.25 (s, 2H), 3.20 – 3.16 (m, 2H), 2.89 – 2.68 (m, 3H), 2.45 – 2.34 (m, 6H), 2.21 (br s, 1H), 2.17 – 2.09 (m, 1H), 1.94 – 1.88 (m, 2H), 1.83 – 1.77 (m, 2H), 1.67 (dp, *J* = 26.3, 7.2 Hz, 4H), 1.49 – 1.41 (m, 2H), 1.38 (t, *J* = 5.6 Hz, 4H), 0.89 (s, 6H) ppm.

**^13^C NMR** (150 MHz, CDCl_3_) δ 171.58 (2C), 169.22, 168.13, 167.82, 167.21, 163.13, 162.33, 157.53, 157.25 (d, *J* = 242.1 Hz), 155.03, 150.95 (dd, *J* = 241.5, 2.2 Hz), 137.39, 134.25, 125.44, 120.36, 119.10 (dd, *J* = 15.6, 4.4 Hz), 118.54 – 118.27 (m), 118.26, 108.75, 105.67 (dd, *J* = 28.3, 2.5 Hz), 80.23, 56.59 (d, *J* = 4.7 Hz), 54.67, 53.42, 52.94, 49.72, 49.30, 47.54, 47.39, 44.81, 41.23, 40.83, 40.14 (d, *J* = 4.9 Hz), 38.53, 35.26 (d, *J* = 8.0 Hz), 32.92, 31.53, 28.34, 26.55, 24.69, 22.78 ppm.

**HRMS-ESI** *m/z* calculated for C_49_H_61_F_2_N_11_O_6_ [M+H]^+^: 938.4774; found 938.4837.

**4c**: 5-(4-(7-((6-(4-(4-((4,4-dimethylpiperidin-1-yl)methyl)-2,5-difluorophenyl)-2-oxo-1,4,9-triazaspiro[5.5]undecan-9-yl)pyrimidin-4-yl)amino)heptanoyl)piperazin-1-yl)-2-(2,6-dioxopiperidin-3-yl)isoindoline-1,3-dione

Compound **4c** was synthesized according to the general procedure described above using 7-((6-(4-(4-((4,4-dimethylpiperidin-1-yl)methyl)-2,5-difluorophenyl)-2-oxo-1,4,9-triazaspiro[5.5]undecan-9-yl)pyrimidin-4-yl)amino)heptanoic acid (**9c**) and 2-(2,6-dioxopiperidin-3-yl)-5-(piperazin-1-yl)isoindoline-1,3-dione hydrochloride (**10a**) at a 26.0 mg scale, yielding 14.0 mg (36.8% yield).

**^1^H NMR** (600 MHz, CDCl_3_) δ 9.67 (s, 1H), 8.12 (s, 1H), 7.69 (d, *J* = 8.5 Hz, 1H), 7.25 (d, *J* = 2.3 Hz, 1H), 7.10 (dd, *J* = 12.9, 6.6 Hz, 1H), 7.03 (dd, *J* = 8.6, 2.4 Hz, 1H), 6.96 (s, 1H), 6.58 (dd, *J* = 10.8, 7.1 Hz, 1H), 5.40 (s, 1H), 5.13 (s, 1H), 4.94 (dd, *J* = 12.4, 5.4 Hz, 1H), 3.85 – 3.75 (m, 2H), 3.74 – 3.70 (m, 4H), 3.65 (t, *J* = 5.3 Hz, 2H), 3.63 – 3.55 (m, 2H), 3.48 (s, 2H), 3.45 – 3.38 (m, 4H), 3.29 – 3.25 (m, 2H), 3.22 – 3.16 (m, 2H), 2.91 – 2.69 (m, 3H), 2.41 (s, 4H), 2.36 (t, *J* = 7.4 Hz, 2H), 2.17 – 2.09 (m, 1H), 1.97 – 1.90 (m, 2H), 1.86 – 1.77 (m, 2H), 1.69 – 1.65 (m, 2H), 1.63 – 1.59 (m, 2H), 1.43 – 1.36 (m, 8H), 0.90 (s, 6H) ppm.

**^13^C NMR** (150 MHz, CDCl_3_) δ 171.72, 171.42, 169.01, 168.04, 167.77, 167.16, 163.17, 162.37, 157.63, 157.25 (d, *J* = 243.8 Hz), 155.06, 151.92 – 149.98 (m), 137.38, 134.28, 125.43, 120.38, 119.21, 118.46 (d, *J* = 6.6 Hz), 118.31, 108.76, 105.82 – 105.41 (m), 80.36, 56.53 (d, *J* = 5.0 Hz), 54.66, 53.44, 52.97, 49.73, 49.27, 47.58, 47.43, 44.83, 41.44, 40.81, 40.20 (d, *J* = 5.9 Hz), 38.53, 35.28 (d, *J* = 5.7 Hz), 32.92, 31.51, 28.89, 28.34, 26.60, 24.97, 22.79 ppm.

**HRMS-ESI** *m/z* calculated for C_50_H_63_F_2_N_11_O_6_ [M+H]^+^: 952.4931; found 952.4998.

**4d**: 5-(4-(8-((6-(4-(4-((4,4-dimethylpiperidin-1-yl)methyl)-2,5-difluorophenyl)-2-oxo-1,4,9-triazaspiro[5.5]undecan-9-yl)pyrimidin-4-yl)amino)octanoyl)piperazin-1-yl)-2-(2,6-dioxopiperidin-3-yl)isoindoline-1,3-dione

Compound **4d** was synthesized according to the general procedure described above using 8-((6-(4-(4-((4,4-dimethylpiperidin-1-yl)methyl)-2,5-difluorophenyl)-2-oxo-1,4,9-triazaspiro[5.5]undecan-9-yl)pyrimidin-4-yl)amino)octanoic acid (**9d**) and 2-(2,6-dioxopiperidin-3-yl)-5-(piperazin-1-yl)isoindoline-1,3-dione hydrochloride (**10a**) at a 20.0 mg scale, yielding 15.0 mg (51.7% yield).

**^1^H NMR** (600 MHz, CDCl_3_) δ 9.61 (br s, 1H), 8.07 (s, 1H), 7.68 (d, *J* = 8.4 Hz, 1H), 7.25 (d, *J* = 2.3 Hz, 1H), 7.10 (dd, *J* = 12.8, 6.5 Hz, 1H), 7.03 (dd, *J* = 8.6, 2.3 Hz, 1H), 6.99 (s, 1H), 6.58 (dd, *J* = 10.8, 7.1 Hz, 1H), 5.39 (s, 1H), 5.07 (s, 1H), 4.92 (dd, *J* = 12.4, 5.4 Hz, 1H), 3.78 (t, *J* = 5.4 Hz, 2H), 3.74 – 3.67 (m, 4H), 3.65 (t, *J* = 5.3 Hz, 2H), 3.63 – 3.51 (m, 2H), 3.49 (s, 2H), 3.46 – 3.38 (m, 4H), 3.28 – 3.24 (m, 2H), 3.14 (t, *J* = 6.9 Hz, 2H), 2.88 – 2.67 (m, 3H), 2.42 (s, 4H), 2.35 (t, *J* = 7.5 Hz, 2H), 2.14 – 2.07 (m, 1H), 1.93 – 1.89 (m, 2H), 1.85 – 1.77 (m, 2H), 1.66 – 1.61 (m, 2H), 1.60 – 1.58 (m, 2H), 1.42 – 1.32 (m, 10H), 0.89 (s, 6H) ppm.

**^13^C NMR** (150 MHz, CDCl_3_) δ 171.80, 171.32, 168.97, 168.02, 167.75, 167.14, 163.19, 162.39, 157.65, 157.25 (d, *J* = 242.6 Hz), 155.06, 150.97 (dd, *J* = 241.5, 2.2 Hz), 137.36, 134.28, 125.45, 120.38, 119.27, 118.54 – 118.38 (m), 118.29, 108.79, 105.68 (dd, *J* = 28.0, 2.4 Hz), 80.24 (br s), 56.51 (d, *J* = 4.5 Hz), 54.69, 53.45, 52.97, 49.74, 49.25, 47.60, 47.45, 44.85, 41.53, 40.80, 40.17 (d, *J* = 7.9 Hz), 38.55, 35.31 (d, *J* = 4.2 Hz), 33.09, 31.50, 29.08, 28.91, 28.35, 26.64, 25.03, 22.79 ppm.

**HRMS-ESI** *m/z* calculated for C_51_H_65_F_2_N_11_O_6_ [M+H]^+^: 966.5087; found 966.5154.

**4e**: 5-(4-(9-((6-(4-(4-((4,4-dimethylpiperidin-1-yl)methyl)-2,5-difluorophenyl)-2-oxo-1,4,9-triazaspiro[5.5]undecan-9-yl)pyrimidin-4-yl)amino)nonanoyl)piperazin-1-yl)-2-(2,6-dioxopiperidin-3-yl)isoindoline-1,3-dione

Compound **4e** was synthesized according to the general procedure described above using 9-((6-(4-(4-((4,4-dimethylpiperidin-1-yl)methyl)-2,5-difluorophenyl)-2-oxo-1,4,9-triazaspiro[5.5]undecan-9-yl)pyrimidin-4-yl)amino)nonanoic acid (**9e**) and 2-(2,6-dioxopiperidin-3-yl)-5-(piperazin-1-yl)isoindoline-1,3-dione hydrochloride (**10a**) at a 10.0 mg scale, yielding 4.0 mg (27.2% yield).

**^1^H NMR** (600 MHz, CDCl_3_) δ 9.25 (s, 1H), 8.13 (s, 1H), 7.70 (d, *J* = 8.5 Hz, 1H), 7.27 (s, 1H), 7.11 (dd, *J* = 12.8, 6.6 Hz, 1H), 7.05 (dd, *J* = 8.5, 2.3 Hz, 1H), 6.70 (s, 1H), 6.58 (dd, *J* = 10.8, 7.1 Hz, 1H), 5.40 (s, 1H), 5.00 (s, 1H), 4.94 (dd, *J* = 12.5, 5.4 Hz, 1H), 3.81 (t, *J* = 5.3 Hz, 2H), 3.73 (s, 2H), 3.71 – 3.64 (m, 4H), 3.60 (m, 2H), 3.49 (s, 2H), 3.47 – 3.39 (m, 4H), 3.31 – 3.22 (m, 2H), 3.20 – 3.14 (m, 2H), 2.92 – 2.69 (m, 3H), 2.41 (s, 4H), 2.37 (t, *J* = 7.5 Hz, 2H), 2.16 – 2.09 (m, 1H), 1.99 – 1.91 (m, 2H), 1.86 – 1.78 (m, 4H), 1.68 – 1.62 (m, 2H), 1.62 – 1.57 (m, 2H), 1.41 – 1.32 (m, 10H), 0.91 (s, 6H) ppm.

**^13^C NMR** (150 MHz, CDCl_3_) δ 171.85, 171.02, 168.78, 167.86, 167.72, 167.10, 163.21, 162.41, 157.73, 157.29 (d, *J* = 238.7 Hz), 155.07, 150.32 – 149.76 (m), 137.30, 134.30, 125.46, 120.40, 119.55, 118.30, 118.29 (d, *J* = 10.7 Hz), 108.77, 105.69 (dd, *J* = 23.8, 2.9 Hz), 80.36, 56.55, 54.68, 53.47, 53.01, 49.76, 49.25, 47.60, 47.48, 44.86, 41.55, 40.76, 40.36 – 39.83 (m), 38.61, 35.33 (d, *J* = 3.4 Hz), 33.10, 31.49, 29.12, 29.10, 28.93, 28.35, 26.75, 25.16, 22.79 ppm.

**HRMS-ESI** *m/z* calculated for C_52_H_67_F_2_N_11_O_6_ [M+H]^+^: 980.5244; found 980.5314.

**4f**: 5-(4-(10-((6-(4-(4-((4,4-dimethylpiperidin-1-yl)methyl)-2,5-difluorophenyl)-2-oxo-1,4,9-triazaspiro[5.5]undecan-9-yl)pyrimidin-4-yl)amino)decanoyl)piperazin-1-yl)-2-(2,6-dioxopiperidin-3-yl)isoindoline-1,3-dione

Compound **4f** was synthesized according to the general procedure described above using 10-((6-(4-(4-((4,4-dimethylpiperidin-1-yl)methyl)-2,5-difluorophenyl)-2-oxo-1,4,9-triazaspiro[5.5]undecan-9-yl)pyrimidin-4-yl)amino)decanoic acid (**9f**) and 2-(2,6-dioxopiperidin-3-yl)-5-(piperazin-1-yl)isoindoline-1,3-dione hydrochloride (**10a**) at a 6.7 mg scale, yielding 4.0 mg (40.2% yield).

**^1^H NMR** (600 MHz, CDCl_3_) δ 9.30 (s, 1H), 8.12 (d, *J* = 0.8 Hz, 1H), 7.70 (d, *J* = 8.5 Hz, 1H), 7.27 (d, *J* = 2.5 Hz, 1H), 7.10 (dd, *J* = 12.8, 6.5 Hz, 1H), 7.05 (dd, *J* = 8.5, 2.4 Hz, 1H), 6.75 (s, 1H), 6.58 (dd, *J* = 10.8, 7.1 Hz, 1H), 5.40 (d, *J* = 1.0 Hz, 1H), 5.02 (s, 1H), 4.94 (dd, *J* = 12.5, 5.4 Hz, 1H), 3.81 (t, *J* = 5.5 Hz, 2H), 3.73 (s, 2H), 3.71 – 3.65 (m, 4H), 3.64 – 3.56 (m, 2H), 3.48 (s, 2H), 3.46 – 3.40 (m, 4H), 3.27 (d, *J* = 4.9 Hz, 2H), 3.16 (q, *J* = 6.6 Hz, 2H), 2.92 – 2.69 (m, 3H), 2.41 (s, 4H), 2.37 (t, *J* = 7.5 Hz, 2H), 2.16 – 2.09 (m, 1H), 1.99 – 1.90 (m, 2H), 1.90 – 1.87 (m, 3H), 1.87 – 1.78 (m, 2H), 1.68 – 1.62 (m, 2H), 1.62 – 1.57 (m, 2H), 1.39 (t, *J* = 5.7 Hz, 5H), 1.31 (s, 6H), 0.90 (s, 6H) ppm.

**^13^C NMR** (150 MHz, CDCl_3_) δ 171.88, 171.06, 168.78, 167.86, 167.72, 167.10, 163.21, 162.42, 157.70, 157.23 (d, *J* = 243.1 Hz), 155.07, 150.99 (d, *J* = 243.5 Hz), 137.31, 134.30, 125.45, 120.39, 119.40 (br s), 118.38 (d, *J* = 10.4 Hz), 118.29, 108.78, 105.69 (dd, *J* = 27.9, 3.1 Hz), 80.31, 56.53 (d, *J* = 5.6 Hz), 54.68, 53.46, 53.02, 49.75, 49.24, 47.62, 47.48, 44.86, 41.59, 40.79, 40.19 (d, *J* = 8.0 Hz), 38.61, 35.35, 33.11, 31.49, 29.19, 29.17, 29.13, 29.07, 28.36, 26.81, 25.18, 22.78 ppm.

**HRMS-ESI** *m/z* calculated for C_53_H_69_F_2_N_11_O_6_ [M+H]^+^: 994.5400; found 994.5460.

**4g**: 3-(5-(4-(7-((6-(4-(4-((4,4-dimethylpiperidin-1-yl)methyl)-2,5-difluorophenyl)-2-oxo-1,4,9-triazaspiro[5.5]undecan-9-yl)pyrimidin-4-yl)amino)heptanoyl)piperazin-1-yl)-1-oxoisoindolin-2-yl)piperidine-2,6-dione

Compound **4g** was synthesized according to the general procedure described above using 7-((6-(4-(4-((4,4-dimethylpiperidin-1-yl)methyl)-2,5-difluorophenyl)-2-oxo-1,4,9-triazaspiro[5.5]undecan-9-yl)pyrimidin-4-yl)amino)heptanoic acid (**9c**) and 3-(1-oxo-5-(piperazin-1-yl)isoindolin-2-yl)piperidine-2,6-dione (**10b**) at a 10.0 mg scale, yielding 1.8 mg (12.0% yield).

**^1^H NMR** (600 MHz, CDCl_3_) δ 8.70 (s, 1H), 8.12 (d, *J* = 0.9 Hz, 1H), 7.75 (d, *J* = 8.6 Hz, 1H), 7.15 (s, 1H), 6.98 (dd, *J* = 8.6, 2.2 Hz, 1H), 6.88 (d, *J* = 2.1 Hz, 1H), 6.62 – 6.56 (m, 2H), 5.42 (d, *J* = 1.0 Hz, 1H), 5.20 (dd, *J* = 13.3, 5.1 Hz, 1H), 5.15 (s, 1H), 4.42 (d, *J* = 15.7 Hz, 1H), 4.27 (d, *J* = 15.7 Hz, 1H), 3.82 – 3.77 (m, 2H), 3.73 (s, 4H), 3.65 (t, *J* = 5.3 Hz, 2H), 3.62 – 3.56 (m, 2H), 3.53 (s, 2H), 3.34 – 3.30 (m, 2H), 3.31 – 3.24 (m, 4H), 3.20 (q, *J* = 6.5 Hz, 2H), 2.94 – 2.87 (m, 1H), 2.87 – 2.78 (m, 1H), 2.46 (s, 3H), 2.38 (t, *J* = 7.4 Hz, 2H), 2.35 – 2.29 (m, 1H), 2.24 – 2.18 (m, 1H), 2.00 – 1.93 (m, 1H), 1.85 – 1.80 (m, 2H), 1.72 – 1.60 (m, 4H), 1.44 – 1.40 (m, 8H), 0.92 (s, 6H) ppm.

**HRMS-ESI** *m/z* calculated for C_50_H_65_F_2_N_11_O_5_ [M+H]^+^: 938.5138; found 938.5209.

**4h**: 3-(5-(4-(8-((6-(4-(4-((4,4-dimethylpiperidin-1-yl)methyl)-2,5-difluorophenyl)-2-oxo-1,4,9-triazaspiro[5.5]undecan-9-yl)pyrimidin-4-yl)amino)octanoyl)piperazin-1-yl)-1-oxoisoindolin-2-yl)piperidine-2,6-dione

Compound **4h** was synthesized according to the general procedure described above using 8-((6-(4-(4-((4,4-dimethylpiperidin-1-yl)methyl)-2,5-difluorophenyl)-2-oxo-1,4,9-triazaspiro[5.5]undecan-9-yl)pyrimidin-4-yl)amino)octanoic acid (**9d**) and 3-(1-oxo-5-(piperazin-1-yl)isoindolin-2-yl)piperidine-2,6-dione (**10b**) at a 6.5 mg scale, yielding 4.0 mg (42.0% yield).

**^1^H NMR** (600 MHz, CDCl_3_) δ 9.07 (s, 1H), 8.13 (d, *J* = 0.8 Hz, 1H), 7.73 (d, *J* = 8.5 Hz, 1H), 7.10 (dd, *J* = 12.8, 6.5 Hz, 1H), 6.97 (dd, *J* = 8.5, 2.2 Hz, 1H), 6.88 (d, *J* = 2.1 Hz, 1H), 6.74 (s, 1H), 6.58 (dd, *J* = 10.8, 7.1 Hz, 1H), 5.40 (d, *J* = 1.0 Hz, 1H), 5.20 (dd, *J* = 13.3, 5.1 Hz, 1H), 5.06 (s, 1H), 4.41 (d, *J* = 15.7 Hz, 1H), 4.26 (d, *J* = 15.7 Hz, 1H), 3.83 – 3.74 (m, 2H), 3.72 (s, 4H), 3.65 (t, *J* = 5.2 Hz, 2H), 3.62 – 3.55 (m, 2H), 3.48 (d, *J* = 1.4 Hz, 2H), 3.35 – 3.24 (m, 4H), 3.17 (q, *J* = 6.8 Hz, 2H), 2.93 – 2.86 (m, 1H), 2.89 – 2.79 (m, 1H), 2.41 (s, 4H), 2.37 (t, *J* = 7.5 Hz, 2H), 2.35 – 2.27 (m, 1H), 2.23 – 2.15 (m, 1H), 2.07 – 1.87 (m, 2H), 1.86 – 1.78 (m, 2H), 1.68 – 1.58 (m, 4H), 1.44 – 1.32 (m, 10H), 0.90 (s, 6H) ppm.

**HRMS-ESI** *m/z* calculated for C_51_H_67_F_2_N_11_O_5_ [M+H]^+^: 952.5295; found 952.5356.

**4i**: 3-(5-(4-(9-((6-(4-(4-((4,4-dimethylpiperidin-1-yl)methyl)-2,5-difluorophenyl)-2-oxo-1,4,9-triazaspiro[5.5]undecan-9-yl)pyrimidin-4-yl)amino)nonanoyl)piperazin-1-yl)-1-oxoisoindolin-2-yl)piperidine-2,6-dione

Compound **4i** was synthesized according to the general procedure described above using 9-((6-(4-(4-((4,4-dimethylpiperidin-1-yl)methyl)-2,5-difluorophenyl)-2-oxo-1,4,9-triazaspiro[5.5]undecan-9-yl)pyrimidin-4-yl)amino)nonanoic acid (**9e**) and 3-(1-oxo-5-(piperazin-1-yl)isoindolin-2-yl)piperidine-2,6-dione (**10b**) at a 9.0 mg scale, yielding 3.4 mg (35.2% yield).

**^1^H NMR** (600 MHz, CDCl_3_) δ 8.96 (s, 1H), 8.12 (s, 1H), 7.74 (d, *J* = 8.5 Hz, 1H), 7.11 (dd, *J* = 12.8, 6.5 Hz, 1H), 6.98 (dd, *J* = 8.6, 2.2 Hz, 1H), 6.88 (d, *J* = 2.2 Hz, 1H), 6.70 (s, 1H), 6.58 (dd, *J* = 10.8, 7.1 Hz, 1H), 5.40 (d, *J* = 1.0 Hz, 1H), 5.20 (dd, *J* = 13.3, 5.1 Hz, 1H), 4.41 (d, *J* = 15.7 Hz, 1H), 4.26 (d, *J* = 15.7 Hz, 1H), 3.84 – 3.74 (m, 2H), 3.73 (s, 4H), 3.65 (t, *J* = 5.2 Hz, 2H), 3.64 – 3.56 (m, 2H), 3.49 (s, 2H), 3.37 – 3.24 (m, 4H), 3.16 (q, *J* = 6.6 Hz, 2H), 2.93 – 2.79 (m, 2H), 2.42 (s, 4H), 2.37 (t, *J* = 7.5 Hz, 2H), 2.35 – 2.27 (m, 1H), 2.23 – 2.17 (m, 1H), 2.06 – 1.92 (m, 3H), 1.87 – 1.78 (m, 2H), 1.69 – 1.57 (m, 4H), 1.42 – 1.29 (m, 12H), 0.91 (s, 6H) ppm.

**^13^C NMR** (150 MHz, CDCl_3_) δ 175.86, 171.85, 170.43, 167.89, 164.47, 162.68, 162.50, 162.33, 157.47 (d, *J* = 250.7 Hz), 156.68, 154.00, 150.86 (d, *J* = 242.1 Hz), 147.54, 142.05, 138.23 (br s), 133.23, 123.97, 122.60, 119.20 (br s), 115.81 (br s), 109.00 (d, *J* = 37.9 Hz), 105.62 – 105.28 (m), 80.10, 56.44 (d, *J* = 25.7 Hz), 53.93, 53.37, 53.29, 52.81, 49.40, 46.96, 41.34, 40.59, 40.20, 37.61 (br s), 35.73, 35.23, 31.93, 29.37, 29.09, 28.97, 28.92, 28.75, 28.20, 26.51, 25.26 ppm.

**HRMS-ESI** *m/z* calculated for C_52_H_69_F_2_N_11_O_5_ [M+H]^+^: 966.5451; found 966.5511.

**4j**: 3-(5-(4-(10-((6-(4-(4-((4,4-dimethylpiperidin-1-yl)methyl)-2,5-difluorophenyl)-2-oxo-1,4,9-triazaspiro[5.5]undecan-9-yl)pyrimidin-4-yl)amino)decanoyl)piperazin-1-yl)-1-oxoisoindolin-2-yl)piperidine-2,6-dione

Compound **4j** was synthesized according to the general procedure described above using 10-((6-(4-(4-((4,4-dimethylpiperidin-1-yl)methyl)-2,5-difluorophenyl)-2-oxo-1,4,9-triazaspiro[5.5]undecan-9-yl)pyrimidin-4-yl)amino)decanoic acid (**9f**) and 3-(1-oxo-5-(piperazin-1-yl)isoindolin-2-yl)piperidine-2,6-dione (**10b**) at a 7.0 mg scale, yielding 4.0 mg (40.8% yield).

**^1^H NMR** (600 MHz, CDCl_3_) δ 9.78 (s, 1H), 8.12 (s, 1H), 7.71 (d, *J* = 8.5 Hz, 1H), 7.15 (s, 1H), 7.10 (dd, *J* = 12.9, 6.6 Hz, 1H), 6.95 (dd, *J* = 8.6, 2.1 Hz, 1H), 6.87 (d, *J* = 2.2 Hz, 1H), 6.57 (dd, *J* = 10.9, 7.1 Hz, 1H), 5.39 (s, 1H), 5.25 (s, 1H), 5.19 (dd, *J* = 13.3, 5.1 Hz, 1H), 4.39 (d, *J* = 15.8 Hz, 1H), 4.25 (d, *J* = 15.8 Hz, 1H), 3.80 – 3.73 (m, 2H), 3.70 (s, 2H), 3.64 (t, *J* = 5.2 Hz, 2H), 3.62 – 3.55 (m, 2H), 3.49 (s, 2H), 3.32 (s, 2H), 3.30 – 3.24 (m, 4H), 3.14 (q, *J* = 6.5 Hz, 2H), 2.90 – 2.76 (m, 2H), 2.41 (s, 4H), 2.35 (t, *J* = 7.6 Hz, 2H), 2.33 – 2.24 (m, 1H), 2.20 – 2.10 (m, 1H), 1.96 – 1.90 (m, 2H), 1.86 – 1.78 (m, 2H), 1.65 – 1.57 (m, 4H), 1.41 – 1.33 (m, 4H), 1.34 – 1.25 (m, 10H), 0.89 (s, 6H) ppm.

**^13^C NMR** (150 MHz, CDCl_3_) δ 171.81, 171.50, 170.34, 169.35, 167.94, 163.19, 162.45, 157.65, 157.24 (d, *J* = 242.9 Hz), 154.00, 150.98 (dd, *J* = 241.6, 2.1 Hz), 143.68, 137.35, 125.14, 122.64, 119.28 (br s), 118.36 (dd, *J* = 22.8, 6.5 Hz), 115.90, 108.78, 105.69 (d, *J* = 28.9 Hz), 80.25, 56.47 (d, *J* = 5.1 Hz), 54.69, 53.44, 53.00, 51.74, 49.75, 48.84, 48.42, 46.92, 45.24, 41.63, 41.11, 40.20 (d, *J* = 12.8 Hz), 38.57, 35.32, 33.17, 31.65, 29.27, 29.24, 29.21, 29.13, 28.35, 26.87, 25.28, 23.55 ppm.

**HRMS-ESI** *m/z* calculated for C_53_H_71_F_2_N_11_O_5_ [M+H]^+^: 980.5608; found 980.5669.

**4k**: 3-(5-(4-(10-((6-(4-(4-((4,4-dimethylpiperidin-1-yl)methyl)-2,5-difluorophenyl)-2-oxo-1,4,9-triazaspiro[5.5]undecan-9-yl)pyrimidin-4-yl)amino)decanoyl)piperazin-1-yl)-1-oxoisoindolin-2-yl)-1-methylpiperidine-2,6-dione

Compound **4k** was synthesized according to the general procedure described above using 10-((6-(4-(4-((4,4-dimethylpiperidin-1-yl)methyl)-2,5-difluorophenyl)-2-oxo-1,4,9-triazaspiro[5.5]undecan-9-yl)pyrimidin-4-yl)amino)decanoic acid (**9f**) and 1-methyl-3-(1-oxo-5-(piperazin-1-yl)isoindolin-2-yl)piperidine-2,6-dione (**10c**) at a 14.0 mg scale, yielding 14.0 mg (67% yield).

**^1^H NMR** (600 MHz, CDCl_3_) δ 8.12 (s, 1H), 7.73 (d, *J* = 8.5 Hz, 1H), 7.11 (dd, *J* = 12.7, 6.4 Hz, 1H), 6.97 (dd, *J* = 8.6, 2.2 Hz, 1H), 6.86 (d, *J* = 12.9 Hz, 2H), 6.58 (dd, *J* = 10.8, 7.1 Hz, 1H), 5.42 (s, 1H), 5.17 (dd, *J* = 13.5, 5.0 Hz, 1H), 4.90 (s, 1H), 4.37 (d, *J* = 15.7 Hz, 1H), 4.25 (d, *J* = 15.7 Hz, 1H), 3.79 (t, *J* = 5.4 Hz, 2H), 3.76 – 3.72 (m, 2H), 3.71 (s, 2H), 3.66 – 3.63 (m, 2H), 3.62 – 3.56 (m, 2H), 3.49 (s, 2H), 3.32 (t, *J* = 5.3 Hz, 2H), 3.30 – 3.25 (m, 4H), 3.18 (d, *J* = 6.9 Hz, 2H), 3.17 (s, 3H), 3.00 – 2.94 (m, 1H), 2.89 – 2.82 (m, 1H), 2.41 (s, 4H), 2.36 (t, *J* = 7.6 Hz, 2H), 2.32 – 2.24 (m, 1H), 2.18 – 2.12 (m, 1H), 1.98 – 1.92 (m, 2H), 1.86 – 1.80 (m, 2H), 1.62 (dt, *J* = 25.8, 7.1 Hz, 4H), 1.42 – 1.36 (m, 6H), 1.33 – 1.29 (m, 6H), 0.90 (s, 6H) ppm.

**^13^C NMR** (150 MHz, CDCl_3_) δ 171.78, 171.35, 170.36, 169.43, 167.75, 163.21, 162.44, 157.68, 157.25 (d, *J* = 242.3 Hz), 153.98, 150.98 (dd, *J* = 241.5, 2.1 Hz), 143.71, 137.41, 125.13, 122.85, 119.11 (br s), 118.39 (dd, *J* = 23.3, 6.5 Hz), 115.88, 108.84, 105.67 (dd, *J* = 27.6, 2.9 Hz), 80.37, 56.51 (d, *J* = 5.0 Hz), 54.66, 53.43, 53.02, 52.43, 49.75, 48.83, 48.47, 47.09, 45.21, 41.63, 41.13, 40.23, 38.53, 35.33, 33.24, 32.11, 29.37, 29.29, 29.27, 29.18, 28.35, 27.16, 26.89, 25.23, 22.84 ppm.

**HRMS-ESI** *m/z* calculated for C_54_H_73_F_2_N_11_O_5_ [M+H]^+^: 994.5764; found 994.5829.

**1.4 Scheme for Synthesis of Compounds 5a-5d**

**1.5 Synthesis of Compounds 5a-5d**

To a solution of 1-bromo-4-(chloromethyl)-2,5-difluorobenzene **11** (826 mg, 3.42 mmol) in DMF (6.0 mL, anhydrous) was added ethyl 4-methylpiperidine-4-carboxylate hydrochloride **12** (710 mg, 3.42 mmol) and K_2_CO_3_ (945 mg, 6.84 mmol). The resulting mixture was stirred at 50 °C for 12 hours and then cooled down to room temperature. The reaction mixture was partitioned between water (60 mL) and ethyl acetate (60 mL), and the organic layer was separated. The aqueous layer was extracted with ethyl acetate (3 x 40 mL), and the combined organic layers were washed with brine (120 mL), dried over anhydrous Na_2_SO_4_, filtered, and concentrated under reduced pressure. The resultant residue was purified by silica gel flash column chromatography (100:0 to 20:1 hexane/ethyl acetate) to give the desired product **13** (1.0 g, 78%) as a clear oil.

**^1^H NMR** (600 MHz, CDCl_3_) δ 7.25 (dd, *J* = 8.6, 5.6 Hz, 2H), 4.17 (q, *J* = 7.1 Hz, 2H), 3.47 (s, 2H), 2.67 – 2.63 (m, 2H), 2.20 (t, *J* = 10.8 Hz, 2H), 2.17 – 2.12 (m, 2H), 1.55 – 1.47 (m, 2H), 1.27 (t, *J* = 7.1 Hz, 3H), 1.20 (s, 3H) ppm.

**^13^C NMR** (150 MHz, CDCl_3_) δ 176.90, 156.65 (dd, *J* = 246.2, 2.7 Hz), 155.51 (dd, *J* = 243.4, 2.9 Hz), 126.91, 119.88 (d, *J* = 27.7 Hz), 117.73 (dd, *J* = 24.8, 5.0 Hz), 106.94, 60.39, 54.76, 50.93, 41.23, 34.79, 25.97, 14.24 ppm.

**ESI-MS** *m/z* calculated for C_16_H_21_BrF_2_NO_2_ [M+H]^+^: 376.1; found 375.9.

*tert*-Butyl 2-oxo-1,4,9-triazaspiro[5.5]undecane-9-carboxylate **14** (270 mg, 1.00 mmol) was dissolved in 1,4-dioxane (2.0 mL, anhydrous), and ethyl 1-(4-bromo-2,5-difluorobenzyl)-4-methylpiperidine-4-carboxylate **13** (376 mg, 1.00 mmol) was added. Then, to this solution was added RuPhos-Pd-G4 (85 mg, 0.10 mmol), RuPhos (47 mg, 0.10 mmol) and Cs_2_CO_3_ (390 mg, 1.20 mmol). The reaction was stirred at 110 °C for 48 hours under a nitrogen atmosphere. The reaction mixture was then cooled down to room temperature, filtered through a pad of Celite, and the inorganic cake was washed with ethyl acetate (20 mL). The filtrate was concentrated, and the residue was purified by silica gel flash column chromatography (100:0 to 12:1 dichloromethane/methanol) to give the desired product **15** (348 mg, 69% yield) as an off-white solid.

**^1^H NMR** (600 MHz, CDCl_3_) δ 7.12 (s, 1H), 7.09 (dd, *J* = 12.9, 6.6 Hz, 1H), 6.58 (dd, *J* = 10.8, 7.2 Hz, 1H), 4.15 (q, *J* = 7.1 Hz, 2H), 3.71 (s, 2H), 3.61 – 3.55 (m, 2H), 3.49 – 3.45 (m, 2H), 3.44 (s, 2H), 3.24 (s, 2H), 2.66 – 2.60 (m, 2H), 2.17 – 2.09 (m, 4H), 1.87 (ddd, *J* = 11.5, 7.3, 3.9 Hz, 2H), 1.76 (ddd, *J* = 12.9, 7.8, 4.0 Hz, 2H), 1.52 – 1.48 (m, 2H), 1.48 (s, 9H), 1.25 (t, *J* = 7.1 Hz, 3H), 1.18 (s, 3H) ppm.

**^13^C NMR** (150 MHz, CDCl_3_) δ 176.97, 168.05, 157.18 (dd, *J* = 242.8, 1.6 Hz), 154.54, 150.97 (dd, *J* = 241.4, 2.3 Hz), 137.66 – 136.76 (m), 119.26 (dd, *J* = 17.2, 6.7 Hz), 118.17 (dd, *J* = 23.3, 6.2 Hz), 105.68 (dd, *J* = 27.7, 2.9 Hz), 79.92, 60.33, 56.37 (d, *J* = 4.9 Hz), 54.66, 53.17, 52.95, 50.81, 41.27, 39.54, 35.67, 34.77, 28.42, 26.04, 14.24 ppm.

**ESI-MS** *m/z* calculated for C_29_H_43_F_2_N_4_O_5_ [M+H]^+^: 565.3; found 565.2.

To a solution of tert-butyl 4-(4-((4-(ethoxycarbonyl)-4-methylpiperidin-1-yl)methyl)-2,5-difluorophenyl)-2-oxo-1,4,9-triazaspiro[5.5]undecane-9-carboxylate **15** (572 mg, 1.01 mmol) in MeOH (4.0 mL) was added aqueous HCl solution (37%, 1.2 mL, 14.52 mmol) dropwise at 0 °C. After the addition was completed, the resulting slurry was stirred at room temperature overnight. The precipitated solid was collected by filtration, washed with cold MeOH (1.0 mL), and dried under vacuum to give the desired product **16** (quantitative yield) as an off-white solid, which was utilized in the next step without further purification.

**ESI-MS** *m/z* calculated for C_24_H_35_F_2_N_4_O_3_ [M+H]^+^: 465.3; found 465.6.

Ethyl 1-(2,5-difluoro-4-(2-oxo-1,4,9-triazaspiro[5.5]undecan-4-yl)benzyl)-4-methylpiperidine-4-carboxylate hydrochloride **16** (300 mg, 0.60 mmol) was suspended in a microwave reaction tube in isopropanol (2.0 mL). 4,6-difluoropyrimidine (105 µL, 1.24 mmol) was added, followed by DIPEA (523 µL, 3.00 mmol). The resulting mixture was stirred at 80 °C under microwave irradiation for three hours. The resultant mixture was then cooled to room temperature and concentrated under reduced pressure. The resulting solid was partitioned between ethyl acetate (25 mL) and saturated NH_4_Cl solution (25 mL) and separated. The organic layer was washed with brine (3 x 25 mL), dried over anhydrous Na_2_SO_4_, filtered, and concentrated. The residue was purified by silica gel flash column chromatography (100:0 to 20:1 dichloromethane/methanol) to give the desired product **17** (310 mg, 89% yield) as an off-white solid.

**^1^H NMR** (600 MHz, CDCl_3_) δ 8.37 (d, *J* = 2.4 Hz, 1H), 7.12 (dd, *J* = 12.9, 6.6 Hz, 1H), 6.61 (s, 1H), 6.63 – 6.59 (m, 1H), 6.07 (s, 1H), 4.17 (q, *J* = 7.1 Hz, 2H), 3.88 – 3.81 (m, 2H), 3.75 (s, 2H), 3.76 – 3.70 (m, 2H), 3.46 (s, 2H), 3.32 (s, 2H), 2.67 – 2.62 (m, 2H), 2.21 – 2.11 (m, 4H), 2.03 (ddd, *J* = 11.6, 7.3, 3.8 Hz, 2H), 1.88 (ddd, *J* = 13.0, 7.9, 4.0 Hz, 2H), 1.50 (ddd, *J* = 13.7, 10.3, 3.6 Hz, 2H), 1.27 (t, *J* = 7.1 Hz, 3H), 1.20 (s, 3H) ppm.

**^13^C NMR** (150 MHz, CDCl_3_) δ 176.97, 172.15, 170.53, 167.77, 164.65 (d, *J* = 10.6 Hz), 158.39 (d, *J* = 17.8 Hz), 158.06 – 156.32 (m), 151.00 (dd, *J* = 241.0, 2.3 Hz), 137.25 – 137.02 (m), 119.62 (dd, *J* = 17.0, 7.0 Hz), 118.22 (dd, *J* = 23.3, 6.3 Hz), 105.77 (dd, *J* = 27.9, 2.8 Hz), 85.37 (d, *J* = 35.7 Hz), 60.36, 56.50 (d, *J* = 4.9 Hz), 54.65, 53.34, 53.01, 50.84, 41.28, 40.44, 35.32, 34.78, 14.24 ppm.

**ESI-MS** *m/z* calculated for C_28_H_36_F_3_N_6_O_3_ [M+H]^+^: 561.3; found 561.1.

To a microwave reaction tube was added ethyl 1-(2,5-difluoro-4-(9-(6-fluoropyrimidin-4-yl)-2-oxo-1,4,9-triazaspiro[5.5]undecan-4-yl)benzyl)-4-methylpiperidine-4-carboxylate **17** (285 mg, 0.51 mmol) and methylamine (5.0 mL, 33% in ethanol). The tube was sealed, and the mixture was stirred at 130 °C under microwave irradiation for one hour. The reaction mixture was cooled to room temperature and concentrated under reduced pressure. The residue was purified by silica gel flash column chromatography (100:0 to 9:1 dichloromethane/methanol) to give the desired product **18** (220 mg, 77% yield) as an off-white solid.

**^1^H NMR** (600 MHz, CDCl_3_) δ 8.17 (s, 1H), 7.12 (dd, *J* = 12.8, 6.6 Hz, 1H), 6.86 (s, 1H), 6.60 (dd, *J* = 10.8, 7.2 Hz, 1H), 5.45 (s, 1H), 4.94 (s, 1H), 4.16 (q, *J* = 7.1 Hz, 2H), 3.80 – 3.75 (m, 2H), 3.74 (s, 2H), 3.68 – 3.61 (m, 2H), 3.46 (s, 2H), 3.30 (s, 2H), 2.91 (d, *J* = 5.2 Hz, 3H), 2.65 (d, *J* = 11.4 Hz, 2H), 2.18 (t, *J* = 10.7 Hz, 2H), 2.16 – 2.11 (m, 2H), 1.99 (ddd, *J* = 11.4, 7.2, 3.7 Hz, 2H), 1.86 (ddd, *J* = 12.8, 7.8, 3.9 Hz, 2H), 1.50 (ddd, *J* = 13.7, 10.7, 3.6 Hz, 2H), 1.26 (t, *J* = 7.1 Hz, 3H), 1.19 (s, 3H) ppm.

**^13^C NMR** (150 MHz, CDCl_3_) δ 176.94, 167.70, 164.03, 162.49, 157.68, 157.21 (dd, *J* = 244.2, 1.7 Hz), 150.74 (dd, *J* = 241.7, 2.2 Hz), 137.63 – 137.33 (m), 118.38 – 118.15 (m), 117.87 (dd, *J* = 30.7, 3.6 Hz), 105.74 (dd, *J* = 28.2, 2.9 Hz), 80.08, 60.37, 56.51 (d, *J* = 4.5 Hz), 54.62, 53.46, 53.36, 53.03, 50.81, 41.26, 40.25, 35.38, 34.71, 28.50, 14.24 ppm.

**ESI-MS** *m/z* calculated for C_29_H_40_F_2_N_7_O_3_ [M+H]^+^: 572.3; found 572.1.

To a solution of ethyl 1-(2,5-difluoro-4-(9-(6-(methylamino)pyrimidin-4-yl)-2-oxo-1,4,9-triazaspiro[5.5]undecan-4-yl)benzyl)-4-methylpiperidine-4-carboxylate **18** (110 mg, 0.19 mmol) in a 1:1 solution of THF/MeOH (1.0 mL) was added 2 M aqueous sodium hydroxide (1.0 mL, 2 mmol). This mixture was heated to 70 °C until fully dissolved and then stirred at 60 °C overnight. Upon reaction completion, monitored by TLC, the base was neutralized with the addition of 3 M HCl, and the reaction mixture was concentrated under reduced pressure. The resultant solid was rinsed with MeOH (5.0 mL), and the suspension was sonicated, followed by filtration. The filtrate was concentrated under reduced pressure to afford the product **19** (quantitative yield) as an off-white solid.

**^1^H NMR** (600 MHz, MeOD) δ 8.00 (s, 1H), 7.35 (dd, *J* = 11.9, 6.7 Hz, 1H), 7.15 (dd, *J* = 11.9, 7.3 Hz, 1H), 5.69 (s, 1H), 4.15 (s, 2H), 4.00 – 3.93 (m, 1H), 3.85 (s, 2H), 3.79 (d, *J* = 12.0 Hz, 2H), 3.61 – 3.56 (m, 2H), 3.50 – 3.42 (m, 1H), 3.29 (d, *J* = 10.3 Hz, 2H), 2.99 – 2.93 (m, 2H), 2.85 (s, 3H), 2.28 (d, *J* = 13.2 Hz, 2H), 1.89 – 1.79 (m, 4H), 1.68 (t, *J* = 11.6 Hz, 2H), 1.23 (s, 3H) ppm.

**^13^C NMR** (150 MHz, MeOD) δ 177.87, 163.31, 161.72, 157.94 (d, *J* = 243.3 Hz), 156.19, 150.19 (d, *J* = 240.7 Hz), 141.92 – 141.50 (m), 120.38 (dd, *J* = 25.5, 5.4 Hz), 109.86 – 109.32 (m), 107.28 (dd, *J* = 28.0, 3.8 Hz), 72.05, 62.29, 60.75, 58.59 (d, *J* = 6.1 Hz), 56.01, 52.44, 50.20, 40.74, 39.34, 34.43, 32.63, 31.55, 26.95 ppm.

**ESI-MS** *m/z* calculated for C_27_H_36_F_2_N_7_O_3_ [M+H]^+^: 544.3; found 544.3.

**General Procedure**: To a solution of 1-(2,5-difluoro-4-(9-(6-(methylamino)pyrimidin-4-yl)-2-oxo-1,4,9-triazaspiro[5.5]undecane-4-yl)benzyl)-4-methylpiperidine-4-carboxylic acid **19** (1 eq) in DMF (0.2 mL) was added HATU (1.2 eq), and the resulting mixture was stirred at room temperature for 30 minutes. A solution of hydrochloride salt **20a-d** (1 eq) and DIPEA (10 eq) in DMF (0.3 mL) was added to the reaction mixture, and the reaction was stirred at room temperature for 30 minutes. The reaction mixture was partitioned between ethyl acetate (10 mL) and water (10 mL), and the layers were separated. The organic layer was washed with brine (10 mL), dried over anhydrous Na_2_SO_4_, filtered and concentrated under reduced pressure. The resulting residue was purified by preparative TLC (8:1 dichloromethane/methanol) to give the desired product **5a-d**.

**5a**: 1-(2,5-difluoro-4-(9-(6-(methylamino)pyrimidin-4-yl)-2-oxo-1,4,9-triazaspiro[5.5]undecan-4-yl)benzyl)-N-(4-((2-(2,6-dioxopiperidin-3-yl)-1,3-dioxoisoindolin-4-yl)amino)butyl)-4-methylpiperidine-4-carboxamide

Compound **5a** was synthesized according to the general procedure described above using 4-((4-aminobutyl)amino)-2-(2,6-dioxopiperidin-3-yl)isoindoline-1,3-dione hydrochloride at a 20 mg scale, yielding 10.7 mg (30.7% yield).

**^1^H NMR** (600 MHz, CDCl_3_) δ 9.20 (s, 1H), 8.17 (s, 1H), 7.51 (dd, *J* = 8.3, 7.3 Hz, 1H), 7.11 (d, *J* = 7.0 Hz, 1H), 7.09 (dd, *J* = 12.7, 6.4 Hz, 1H), 6.91 (d, *J* = 8.6 Hz, 1H), 6.61 (dd, *J* = 10.8, 7.2 Hz, 1H), 6.57 (s, 1H), 6.25 (t, *J* = 5.7 Hz, 1H), 5.71 (t, *J* = 5.7 Hz, 1H), 5.44 (s, 1H), 5.18 (s, 1H), 4.93 (dd, *J* = 12.4, 5.4 Hz, 1H), 3.76 (s, 2H), 3.74 – 3.70 (m, 2H), 3.66 – 3.63 (m, 2H), 3.48 (s, 2H), 3.37 – 3.31 (m, 4H), 3.30 (s, 2H), 2.91 – 2.87 (m, 1H), 2.89 (d, *J* = 5.2 Hz, 3H), 2.86 – 2.70 (m, 2H), 2.59 – 2.54 (m, 2H), 2.39 – 2.33 (m, 2H), 2.17 – 2.11 (m, 1H), 2.04 – 1.93 (m, 4H), 1.87 – 1.81 (m, 2H), 1.73 – 1.62 (m, 4H), 1.60 – 1.54 (m, 2H), 1.18 (s, 3H) ppm.

**^13^C NMR** (150 MHz, CDCl_3_) δ 176.98, 171.42, 169.58, 168.72, 167.84, 167.59, 163.89, 162.50, 157.47, 157.26 (d, *J* = 242.7 Hz), 155.43, 150.96 (d, *J* = 239.5 Hz), 146.84, 137.96 – 137.15 (m), 136.20, 132.50, 116.69, 111.63, 110.09, 108.78, 106.19 – 105.49 (m), 90.67, 84.27, 56.52 (d, *J* = 5.0 Hz), 54.52, 52.98, 50.12, 48.92, 42.18, 40.71, 40.26, 38.92, 35.31, 34.77, 31.48, 29.71, 28.48, 27.20, 26.51, 22.84 ppm.

**HRMS-ESI** *m/z* calculated for C_44_H_54_F_2_N_11_O_6_ [M+H]^+^: 870.4148; found 870.4212.

**5b**: 1-(2,5-difluoro-4-(9-(6-(methylamino)pyrimidin-4-yl)-2-oxo-1,4,9-triazaspiro[5.5]undecan-4-yl)benzyl)-N-(6-((2-(2,6-dioxopiperidin-3-yl)-1,3-dioxoisoindolin-4-yl)amino)hexyl)-4-methylpiperidine-4-carboxamide

Compound **5b** was synthesized according to the general procedure described above using 4-((6-aminohexyl)amino)-2-(2,6-dioxopiperidin-3-yl)isoindoline-1,3-dione hydrochloride at a 20 mg scale, yielding 4.4 mg (12.2% yield).

**^1^H NMR** (600 MHz, CDCl_3_) δ 9.10 (s, 1H), 8.17 (s, 1H), 7.51 (dd, *J* = 8.3, 7.3 Hz, 1H), 7.16 (s, 1H), 7.11 (d, *J* = 7.0 Hz, 1H), 6.90 (d, *J* = 8.5 Hz, 1H), 6.61 (dd, *J* = 10.8, 7.2 Hz, 1H), 6.37 (s, 1H), 6.25 (t, *J* = 5.5 Hz, 1H), 5.65 – 5.62 (m, 1H), 5.44 (s, 1H), 5.28 (br s, 1H), 4.93 (dd, *J* = 12.4, 5.4 Hz, 1H), 3.77 (s, 2H), 3.76 – 3.71 (m, 2H), 3.70 – 3.64 (m, 2H), 3.51 (s, 2H), 3.33 – 3.31 (m, 2H), 3.30 – 3.26 (m, 4H), 2.93 – 2.88 (m, 1H), 2.90 (d, *J* = 5.2 Hz, 3H), 2.86 – 2.72 (m, 2H), 2.66 – 2.56 (m, 2H), 2.45 – 2.38 (br s, 2H), 2.18 – 2.13 (m, 1H), 2.04 – 1.96 (m, 4H), 1.86 – 1.81 (m, 2H), 1.72 – 1.67 (m, 2H), 1.65 – 1.58 (m, 2H), 1.57 – 1.51 (m, 2H), 1.50 – 1.43 (m, 2H), 1.42 – 1.37 (m, 2H), 1.19 (s, 3H) ppm.

**^13^C NMR** (150 MHz, CDCl_3_) δ 176.80, 171.33, 169.58, 168.67, 167.61, 162.41, 162.34, 157.35, 158.54 – 156.80 (m), 154.99, 151.67, 146.95, 136.76, 136.13, 132.51, 116.63, 116.60, 116.46, 111.46, 109.95, 106.60, 79.87 – 79.67 (m), 56.41, 55.35, 54.38, 53.71, 52.81, 50.04, 48.92, 42.56, 40.32, 39.37, 35.30, 34.71, 31.94, 29.67, 29.56, 29.37, 29.11, 28.48, 26.61, 22.87 ppm.

**HRMS-ESI** *m/z* calculated for C_46_H_58_F_2_N_11_O_6_ [M+H]^+^: 898.4461; found 898.4531.

**5c**: 1-(2,5-difluoro-4-(9-(6-(methylamino)pyrimidin-4-yl)-2-oxo-1,4,9-triazaspiro[5.5]undecan-4-yl)benzyl)-N-(8-((2-(2,6-dioxopiperidin-3-yl)-1,3-dioxoisoindolin-4-yl)amino)octyl)-4-methylpiperidine-4-carboxamide

Compound **5c** was synthesized according to the general procedure described above using 4-((8-aminooctyl)amino)-2-(2,6-dioxopiperidin-3-yl)isoindoline-1,3-dione hydrochloride at a 20 mg scale, yielding 10.6 mg (28.6% yield).

**^1^H NMR** (600 MHz, CDCl_3_) δ 9.71 (s, 1H), 8.17 (s, 1H), 7.50 (dd, *J* = 8.2, 7.5 Hz, 1H), 7.12 – 7.07 (m, 2H), 6.89 (d, *J* = 8.6 Hz, 1H), 6.86 (s, 1H), 6.60 (dd, *J* = 10.7, 7.2 Hz, 1H), 6.24 (t, *J* = 5.6 Hz, 1H), 5.63 (t, *J* = 5.7 Hz, 1H), 5.43 (s, 1H), 5.34 (s, 1H), 4.93 (dd, *J* = 12.4, 5.4 Hz, 1H), 3.78 – 3.71 (m, 4H), 3.68 – 3.63 (m, 2H), 3.49 (s, 2H), 3.31 – 3.28 (m, 2H), 3.28 – 3.24 (m, 4H), 2.90 – 2.87 (m, 1H), 2.88 (d, *J* = 5.2 Hz, 3H), 2.85 – 2.70 (m, 2H), 2.60 – 2.52 (m, 2H), 2.40 – 2.34 (m, 2H), 2.16 – 2.11 (m, 1H), 2.03 – 1.94 (m, 4H), 1.88 – 1.82 (m, 2H), 1.70 – 1.65 (m, 2H), 1.60 – 1.54 (m, 2H), 1.52 – 1.46 (m, 2H), 1.45 – 1.39 (m, 2H), 1.38 – 1.32 (m, 6H), 1.18 (s, 3H) ppm.

**^13^C NMR** (150 MHz, CDCl_3_) δ 176.66, 171.58, 169.84, 169.59, 168.84, 167.84, 167.67, 166.52, 163.85, 162.51, 157.39, 157.25 (d, *J* = 245.8, 1.6 Hz), 155.10, 151.76, 147.00, 138.62 (d, *J* = 13.6 Hz), 136.09, 132.51, 118.43, 116.65, 111.37, 109.87, 107.48, 90.80, 84.26, 56.54, 54.48, 50.11, 48.91, 42.61, 40.63, 40.26, 39.50, 35.32, 34.76, 31.94, 29.71, 29.64, 29.19, 29.11, 28.48, 26.84, 26.81, 22.89 ppm.

**HRMS-ESI** *m/z* calculated for C_48_H_62_F_2_N_11_O_6_ [M+H]^+^: 926.4774; found 926.4836.

**5d**: 1-(2,5-difluoro-4-(9-(6-(methylamino)pyrimidin-4-yl)-2-oxo-1,4,9-triazaspiro[5.5]undecan-4-yl)benzyl)-N-(10-((2-(2,6-dioxopiperidin-3-yl)-1,3-dioxoisoindolin-4-yl)amino)decyl)-4-methylpiperidine-4-carboxamide

Compound **5d** was synthesized according to the general procedure described above using 4-((10-aminodecyl)amino)-2-(2,6-dioxopiperidin-3-yl)isoindoline-1,3-dione hydrochloride at a 20 mg scale, yielding 17.7 mg (46.4% yield).

**^1^H NMR** (600 MHz, CDCl_3_) δ 10.06 (s, 1H), 8.15 (s, 1H), 7.49 (dd, *J* = 8.5, 7.1 Hz, 1H), 7.16 (s, 1H), 7.11 – 7.06 (m, 2H), 6.88 (d, *J* = 8.6 Hz, 1H), 6.59 (dd, *J* = 10.8, 7.1 Hz, 1H), 6.23 (t, *J* = 5.4 Hz, 1H), 5.65 (t, *J* = 5.6 Hz, 1H), 5.41 (s, 2H), 4.92 (dd, *J* = 12.4, 5.4 Hz, 1H), 3.78 – 3.71 (m, 4H), 3.67 – 3.60 (m, 2H), 3.48 (s, 2H), 3.29 – 3.27 (m, 2H), 3.27 – 3.23 (m, 4H), 2.90 – 2.88 (m, 1H), 2.86 (d, *J* = 5.0 Hz, 3H), 2.84 – 2.70 (m, 2H), 2.59 – 2.54 (m, 2H), 2.39 – 2.32 (m, 2H), 2.15 – 2.11 (m, 1H), 2.03 – 1.98 (m, 2H), 1.98 – 1.92 (m, 2H), 1.87 – 1.82 (m, 2H), 1.66 (p, *J* = 7.1 Hz, 2H), 1.59 – 1.53 (m, 2H), 1.51 – 1.45 (m, 2H), 1.45 – 1.39 (m, 2H), 1.36 – 1.28 (m, 10H), 1.17 (s, 3H) ppm.

**^13^C NMR** (150 MHz, CDCl_3_) δ 176.66, 171.60, 169.57, 168.86, 167.81, 167.67, 163.93, 162.51, 157.51, 158.15 – 156.37 (m), 151.75 – 150.11 (m), 147.01, 143.57, 137.66 – 137.46 (m), 136.09, 132.51, 116.64, 111.33, 109.83, 105.94 – 105.76 (m), 91.00, 79.86, 56.53 (d, *J* = 4.6 Hz), 54.52, 52.95, 50.10, 48.90, 42.66, 40.64, 40.23, 39.50, 35.34, 34.80, 31.49, 29.71, 29.65, 29.39, 29.36, 29.26, 29.18, 28.48, 26.92, 26.86, 22.90 ppm.

**HRMS-ESI** *m/z* calculated for C_50_H_66_F_2_N_11_O_6_ [M+H]^+^: 954.5087; found 954.5151.

**1.6 Biological Methods**

**Cell Lines and Culture Conditions**: The human acute myeloid leukemia (AML) cell lines MOLM13 and MV4.11, as well as the HEK 293T epithelial kidney cell line, were obtained from the American Type Culture Collection (ATCC, USA). AML cells were maintained in RPMI-1640 medium (Gibco, USA) supplemented with 10% fetal bovine serum (FBS) and 1% penicillin-streptomycin (Sigma, Germany). HEK 293T cells were cultured in complete Dulbecco’s Modified Eagle Medium (DMEM) containing 10% FBS and 1% penicillin-streptomycin (Sigma). All cell lines were maintained under standard conditions in a humidified atmosphere at 37 °C with 5% CO₂.

**Cell Viability Assay**: AML cells were seeded in 96-well plates at a density of 5,000 cells per well and treated with varying concentrations of ZW30441 or vehicle (dimethyl sulfoxide, DMSO). Each condition was performed in triplicate. After the treatment period, cell viability was measured using the Cell Counting Kit-8 (CCK-8; APExBIO, USA, Cat# K1018) according to the manufacturer’s instructions. Absorbance was recorded at 450 nm using a SPECTROstar microplate reader (BMG LABTECH, USA).

**Cell Proliferation Assay**: To assess cell growth, 1×10⁵ cells were seeded per well in 24-well plates in triplicate and treated with 5 µM concentrations of the test compounds. The culture medium containing the compounds was refreshed every 48 hours, and cell densities were maintained below 1×10⁶ cells/mL through periodic dilution. Cell numbers were measured every other day over a 4-day period using a DS-11 automated cell counter (DeNovix, USA). MOLM13 and MV4-11 cells were treated with ZW30441 (5 µM) and compared to cells treated with STM2457, UZH2, or vehicle (dimethyl sulfoxide, DMSO).

**Western Blot Analysis**: Following treatment, total cellular proteins were extracted using RIPA buffer (Auragene, Changsha, China). Equal amounts of protein were separated by 12% SDS-PAGE and transferred onto PVDF membranes (Thermo Fisher, USA, LC2002). Membranes were blocked with 5% non-fat dry milk in TBST for 2 hours at room temperature, then incubated overnight at 4 °C with primary antibodies against METTL3 (Abcam, UK, ab195352), METTL14 (Sigma-Aldrich, SAB5700855), and ACTIN (Proteintech, Germany, 23660-1-AP). After washing, membranes were incubated with horseradish peroxidase (HRP)-conjugated secondary antibodies (anti-rabbit or anti-mouse IgG; 1:5000) for 2 hours at room temperature. Protein bands were visualized using an enhanced chemiluminescence (ECL) detection kit (Amersham Pharmacia), following the manufacturer's protocol. Band intensities were quantified using ImageJ software and normalized to ACTIN as a loading control. Data was analyzed using GraphPad Prism 9. Degradation efficiency was evaluated by calculating DC₅₀, the drug concentration required to reduce target protein levels by 50%, and Dₘₐₓ, the maximum level of degradation observed across all tested concentrations.

**Materials for Microsomal Stability:** Reagent-grade KH2PO4, K2HPO4, acetonitrile, methanol, and formic acid (LC-MS grade) were purchased from Fisher Scientific. Phenacetin (≥98% purity) and verapamil (≥98% purity) were obtained from Sigma-Aldrich. Reduced nicotinamide adenine dinucleotide phosphate (NADPH) and human liver microsomes (Lot No # 1210270) were obtained from MP Biomedicals and BioIVT, respectively. Ultrapure water was used for analysis.

**Metabolic Stability**: Metabolic stability was assessed using male human liver microsomes. **ZW30441** was incubated at a concentration of 1 µM with 1 mg/mL of microsomal protein in 50 mM phosphate buffer (pH 7.4). The metabolic reaction was initiated by the addition of NADPH (1 mM) and carried out at 37 °C, 5% CO₂, 120 rpm. Samples were collected at 0, 5, 10, 15, 30 and 60 min to assess compound metabolism over time. Verapamil served as the positive control. The study was performed in triplicate. Reactions were quenched with ice-cold acetonitrile containing phenacetin (5 ng/mL) as an internal standard (IS). Following protein precipitation, the samples were filtered using a Solvinert 96-well plate, centrifuged at 2000 rpm, and analyzed by LC–MS/MS. Area ratios (compound peak area to IS peak area) from each sample were used to calculate the percentage of compound remaining over time, which was then used to determine the elimination rate constant (k) by calculating the slope of the linear regression of the natural logarithm of the remaining compound versus time, following a previously reported protocol [30].

**2. Compound Spectra**

**2.1 NMR Spectra for reported compounds**

Compound **7**

Compound **4a**

Compound **4b**

Compound **4c**

Compound **4d**

Compound **4e**

Compound **4f**

Compound **4g**


Compound **4h**

Compound **4i**

Compound **4j**

Compound **4k**

Compound **13**

Compound **15**

Compound **16**

Compound **17**


Compound **18**

Compound **19**

Compound **5a**

Compound **5b**

Compound **5c**

Compound **5d**

**2.2 HRMS data for final compounds**

**4a**

**4b**

**4c**

**4d**

**4e**

**4f**

**4g**

**4h**

**4i**

**4j**

**4k**

**5a**

**5b**

**5c**

**5d**
